# The adoption paradox for veterinary professionals in China: high use of artificial intelligence despite low familiarity

**DOI:** 10.3389/fvets.2026.1727001

**Published:** 2026-03-11

**Authors:** Shumin Li, Xiaoyun Lai

**Affiliations:** 1 College of Veterinary Medicine, Jilin University, Jilin, China; 2 West East Small Animal Veterinary Conference, Jiangsu, China

**Keywords:** artificial intelligence, China, clinical decision making, survey, technology adoption, veterinary medicine

## Abstract

**Introduction:**

The global integration of artificial intelligence (AI) into veterinary medicine is advancing, yet its adoption in major markets like China remains uncharacterized. This study aimed to provide the first exploratory analysis of AI perception and adoption among veterinary professionals in China.

**Methods:**

A cross-sectional survey was administered to 455 veterinary professionals in China from May to July 2025. Data on AI familiarity, adoption rates, application priorities, and perceived drivers and barriers were analyzed using descriptive statistics and thematic analysis.

**Results:**

We identified a distinct adoption paradox: 71.0% of respondents incorporated AI into their workflow, yet 44.6% of these active users reported low familiarity with the technology. Adoption was primarily practitioner-driven and focused on core clinical tasks, including AI-assisted disease diagnosis (50.1%) and prescription calculation (44.8%). The primary barrier to use was concern about AI reliability and accuracy (54.3%). A strong consensus (93.8%) emerged supporting regulatory oversight of AI by veterinary authorities.

**Discussion:**

The adoption paradox is driven by a practitioner-led, “inside-out” integration model where AI is used to augment clinical capabilities, countered by an “interpretability gap” that limits trust and familiarity. This contrasts with the more administrative, “outside-in” pattern seen in North America. The findings underscore a need for specialized veterinary AI tools, enhanced training focused on critical appraisal, and robust regulatory frameworks to safely harness AI’s potential in one of the world’s largest veterinary markets.

## Introduction

Over the past decade, there has been extensive research in healthcare demonstrating the potential of artificial intelligence (AI) in healthcare through its capacity to analyze vast datasets, identify complex patterns, and automate routine tasks (1, 2). Beyond these programmed functions, AI can also yield unintended yet valuable insights, a phenomenon sometimes termed ‘opportunistic AI’. For instance, analysis of medical images can reveal secondary pathologies beyond the initial scope of the examination (3, 4). In veterinary medicine, the promise of AI has been demonstrated through numerous commercial applications that enhance diagnostics, predictive analytics, patient communication, and personalized medicine (5–12). While the potential of these technologies is widely acknowledged, their practical adoption and impact on clinical decision-making are not yet fully understood, particularly outside of North America and Europe. Evaluating this impact is critical for translating these promising technologies from development into routine clinical practice for both veterinary and human medicine (13).

Recent research, such as the 2024 survey of veterinary professionals in the United States and Canada (14), has provided an initial benchmark, correlating AI familiarity with professional optimism and identifying key implementation barriers in a western context. However, this perspective is geographically limited and leaves a knowledge gap concerning AI adoption in other major international markets. China, with its multi-billion-dollar pet care industry and large network of modern veterinary facilities, represents one of the largest and most dynamic such markets, yet professional attitudes toward and adoption of AI remain completely uncharacterized (15). Understanding the perceptions, priorities, and challenges within this distinct ecosystem is essential for a complete global assessment of AI’s trajectory in the veterinary field.

Therefore, the purpose of this study was to address this knowledge gap by providing the first exploratory analysis of AI perception and adoption among veterinary professionals in China. We hypothesized that the unique demographics, market structure, the legislative frameworks, and technological landscape in China would foster a distinct pattern of AI integration. By characterizing this pattern, we aim to provide crucial insights for technology developers, educators, and regulators seeking to responsibly and effectively support the veterinary profession in China.

## Methods

### Study design and participants

A descriptive, cross-sectional survey design (16) was used to investigate the adoption and perception of AI among veterinary professionals in China. The study population consisted of veterinary personnel, including veterinarians, technicians, and administrative staff. A convenience sampling approach was employed, recruiting participants between May and July 2025 through two primary channels: in-person at a major national veterinary conference [West East Small Animal Conference (WESAC)] and online through professional networks of the Chinese Veterinary Medical Association (CVMA).

Due to the broad distribution methods employed, which included open professional networks and potential snowball sharing, the exact size of the sampling frame and thus a precise response rate cannot be determined. This is a recognized limitation of web-based surveys designed to achieve wide reach across a large, diffuse population. While this prevents a traditional response rate calculation, the final sample of 455 complete responses provides a robust, novel dataset that meets the target sample size for descriptive analysis (yielding a margin of error of approximately ±5% for proportional estimates) and offers the first quantitative insights into AI adoption in this market. Participation was voluntary and anonymous.

### Ethical considerations

This study was conducted in accordance with the ethical principles of the Declaration of Helsinki and international standards for survey research. As the research involved a one-time, anonymous, and voluntary survey of adult professionals regarding their workplace technology use, it was determined to be exempt from formal Institutional Review Board (IRB) approval. All participants were provided with an introductory statement explaining the study’s purpose, the anonymity of the data, and the voluntary nature of their involvement. Completion and submission of the online survey constituted informed consent.

### Survey instrument

The data collection instrument was a 26-item questionnaire administered in Mandarin via a commercial online survey platform (Wenjuanxing). The questionnaire was adapted from a previously published 2024 North American (NA) survey (14) to facilitate international contextualization. The adaptation process involved two key steps: (1) Translation and Cultural Validation: The original English questionnaire was translated into Mandarin using a bilingual veterinary professional and generative AI tools to optimize linguistic clarity. To ensure this AI-assisted process did not affect content validity, the instrument was back-translated by an independent translator to confirm conceptual equivalence. (2) Contextual Modification: While the core structure and the majority of questions were retained to allow for contextual comparison, modifications were made to reflect the Chinese market. This included adding examples of AI tools prevalent in China (e.g., Deepseek, DouBao) to the options for Q9 and adjusting demographic categories (e.g., institutional types) to align with the Chinese veterinary landscape. The final instrument included 21 categorical (single or multiple-choice) and 5 open-ended questions, structured into four sections: (1) demographic and professional information; (2) familiarity and usage of AI tools; (3) perceptions, attitudes, and trust in AI; and (4) qualitative feedback. The full questionnaire (original and translated versions) is provided in the Supplementary material. The primary aim of both the original and adapted surveys was exploratory description and contextual benchmarking; as such, formal psychometric validation (e.g., reliability testing) was not conducted.

A limitation of the survey instrument is the potential ambiguity in Question 8 (“Which AI Tools or Fields Have You Known or Used?”), which conflates awareness with actual use. Furthermore, the combination of “diagnosis” and “treatment” into a single option and the broad scope of “Drug doses and prescription” may limit the granular interpretation of these specific application areas. These limitations are acknowledged and will be considered in the interpretation of the results.

### Statistical analysis

All quantitative data were analyzed using Python (3.8.8). Response distributions for all categorical variables were first assessed for non-random patterns using chi-square goodness-of-fit tests. Self-reported variables, such as AI familiarity, were treated as ordinal constructs based on their Likert-scale format, consistent with standard practice for descriptive analysis of survey data in similar technological adoption studies. To evaluate the relationships between key variable pairs (e.g., AI familiarity and AI adoption rate), chi-square tests of independence were performed, a method appropriate for analyzing associations between two categorical variables. For all statistically significant associations, Cramér’s *V* was calculated to measure the effect size and determine the strength of the relationship. A *p*-value of < 0.001 was set as the statistical significance threshold for all analyses to control for the increased risk of Type I errors due to multiple comparisons. Qualitative data from open-ended questions were analyzed thematically.

### Contextual comparison with north American data

To situate the findings from China within the global conversation, a descriptive comparison was made with the published summary results of the 2024 NA survey of veterinary professionals (*n* = 3,968) (14). It is critical to note that this was not a primary aim of the study and that the comparison has inherent constraints. The two surveys were conducted a year apart, used different sampling frames, and most importantly, the professional composition of the cohorts differed fundamentally (e.g., 24.3% veterinarians in NA vs. 81.5% in China). Due to these factors and the lack of access to the raw NA dataset, which prevents adjustment for confounding variables, formal inferential statistical testing between the two cohorts was not performed. Therefore, the comparison is presented as a descriptive overview to highlight potential trends and contextual contrasts, and any numerical differences should be interpreted with caution.

## Results

### Demographics and professional profile of the Chinese cohort

**Figure 1:**
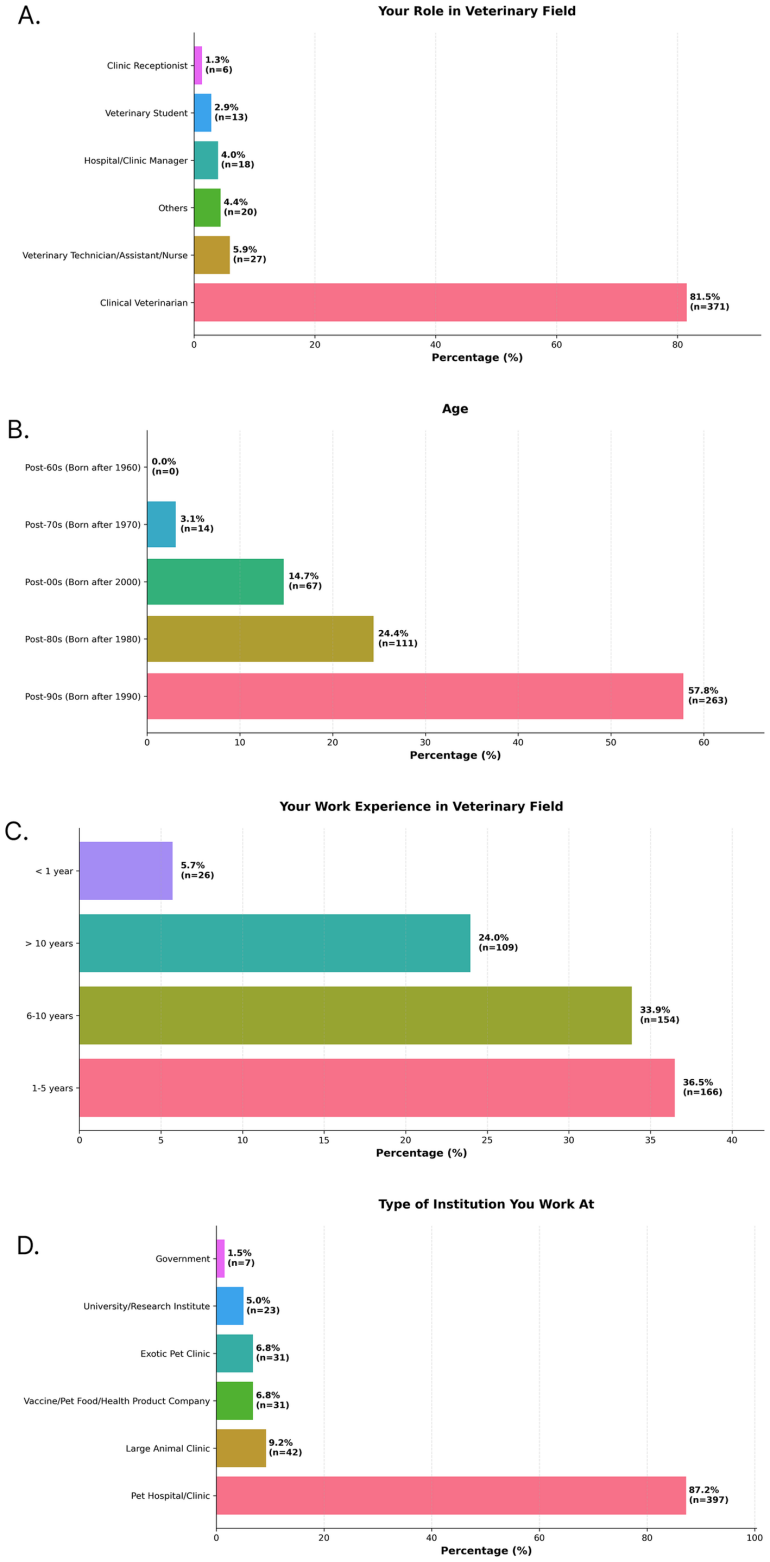
Demographic characteristics of study participants. Bar charts show the distribution of responses to four survey questions: **(A)** your role in veterinary field, **(B)** age, **(C)** your work experience in veterinary field, and **(D)** Type of institution you work at. Percentages for each category are displayed on the bars, along with the number of respondents (*n*) for that category.

A total of 455 valid responses were collected from veterinary professionals in China. The respondent pool consisted primarily of frontline clinicians, with 81.5% identifying as veterinarians (Figure 1A). Other roles represented included veterinary technicians, assistants, or nurses (5.9%), hospital or clinic managers (4.0%), and veterinary students (2.9%). The cohort was composed of early-to-mid-career professionals. The largest age group was born in the 1990s (57.8%, Figure 1B), followed by those born in the 1980s (24.4%). This aligns with reported professional experience, where 36.5% had 1–5 years of experience and 33.8% had 6–10 years of experience (Figure 1C). The majority of participants (87.2%, Figure 1D) worked in small animal pet hospitals or clinics.

### Data analysis

**Figure 2:**
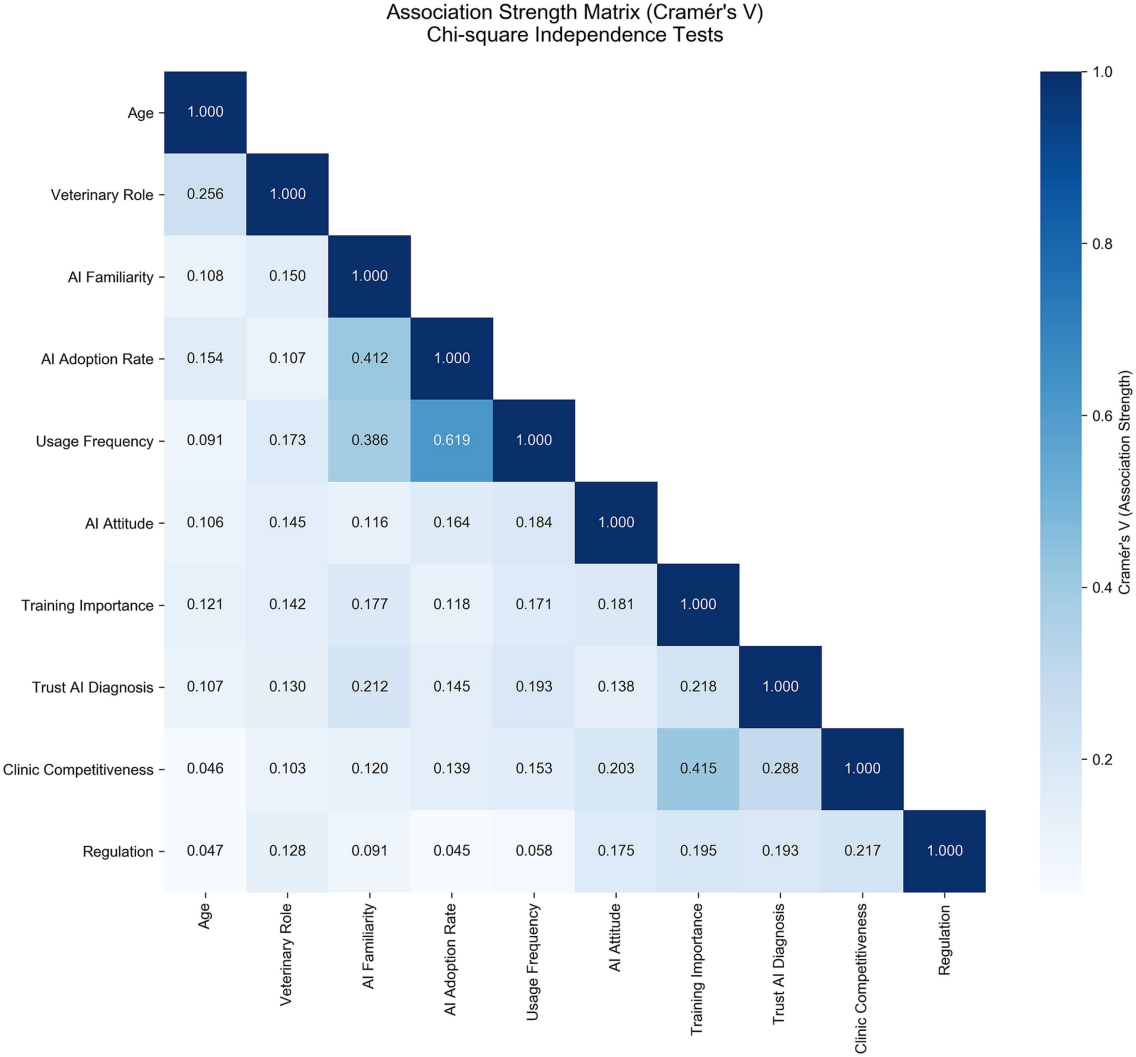
Heatmap illustrating the association strength between key categorical variables using Cramér’s *V* from Chi-square tests of independence. The intensity of the blue color and the numerical value in each cell represent the effect size of the association between the corresponding pair of variables. Of the 45 variable pairs tested, 18 (40%) demonstrated a statistically significant association (*p* < 0.001). The most notable strong associations are between AI adoption rate and usage frequency (*V* = 0.619), Training importance and clinic competitiveness (*V* = 0.415), and AI familiarity and AI adoption rate (*V* = 0.412).

All 21 categorical variables in the Chinese survey demonstrated a significant deviation from a uniform distribution (*p* < 0.001). Chi-square tests of independence on key variable pairs within the cohort revealed that 18 of 45 pairs (40%) had a statistically significant association (*p* < 0.001). The three strongest associations, based on effect size, were between AI Familiarity and AI Adoption Rate (Cramér’s *V* = 0.412), AI Adoption Rate and Usage Frequency (Cramér’s *V* = 0.619), and perceived Importance of Training and perceived increase in clinic competitiveness from AI (Cramér’s *V* = 0.415). (Figure 2). Full visualizations for all response distributions are available in the Supplementary material.

### AI familiarity, adoption, and usage in China

**Figure 3:**
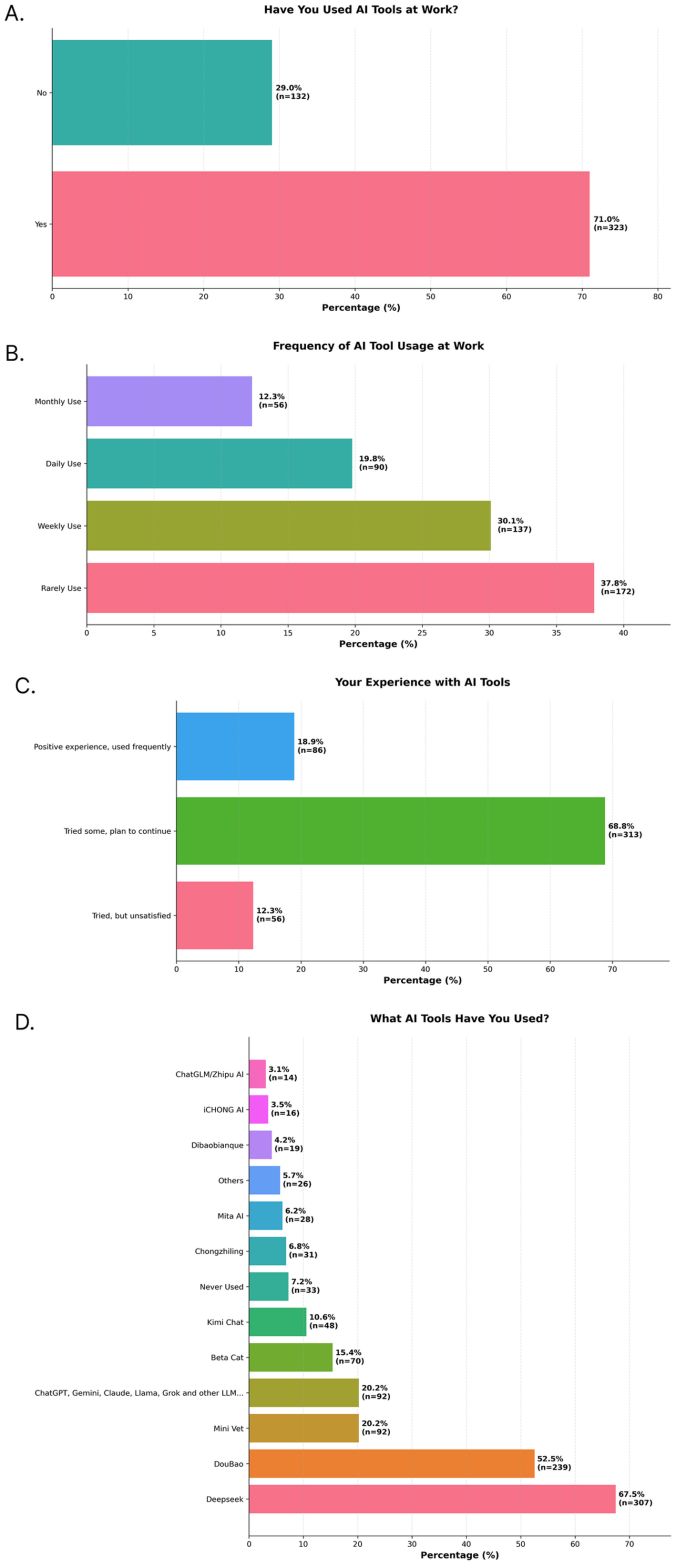
Survey results that highlight AI familiarity, adoption, and usage patterns in China. Bar charts show the distribution of responses to five survey questions: **(A)** Have you used AI tools in veterinary practice, **(B)** Frequency of AI tool usage at work, **(C)** Your experience with AI tools, and **(D)** What AI tools have you used? Percentages for each category are displayed on the bars, along with the number of respondents (*n*) for that category.

Most Chinese respondents reported low-to-moderate familiarity with AI, with 51.2% describing themselves as “not very familiar” and 4.2% as “completely unfamiliar.” Only 9.7% felt “very familiar” and 35.0% felt “somewhat familiar.” Despite this, 71.0% of respondents confirmed they have used AI tools in their professional work (Figure 3A). Among AI users (Figure 3B), 19.8% reported using the tools daily and 30.1% used them weekly. Regarding user experience (Figure 3C), 68.8% stated they have “tried some [tools] and are prepared to continue exploring,” while 18.9% reported that the tools work well and are used regularly. Usage was dominated by Chinese/locally serviced large language models (LLMs) (Figure 3D), including Deepseek (67.5%) and Doubao (52.5%), while internationally recognized models like ChatGPT and Gemini (20.2%) and veterinary-specific platforms such as MiniVet (20.2%) were also used.

The relationship between user status and self-reported familiarity is detailed in Table 1. It shows that adoption rates are highest among those with high familiarity (88.2%), but a substantial number of users (144 out of 323, or 44.6%) report low familiarity (‘not very familiar’ or ‘completely unfamiliar’), directly illustrating the coexistence of high use and low understanding.

**Table 1:** Cross-tabulation of AI user status against self-reported familiarity levels (*n* = 455).

Familiarity level	Total (*n* = 455)	Users (*n* = 323)	Non-users (*n* = 132)	Adoption rate within group
High familiarity	**203**	**179**	**24**	**88.2%**
Very familiar	44	42	2	95.5%
Somewhat familiar	159	137	22	86.2%
Low familiarity	**252**	**144 (44.6%)**	**108**	**57.1%**
Not very familiar	233	138	95	59.2%
Completely unfamiliar	19	6	13	31.6%
Total	455	323 (100%)	132 (100%)	71.0%

Bolded rows represent the aggregate totals for High familiarity (comprising “Very” and “Somewhat”) and Low familiarity (comprising “Not very” and “Completely unfamiliar”).

### Primary applications and perceptions in China

**Figure 4:**
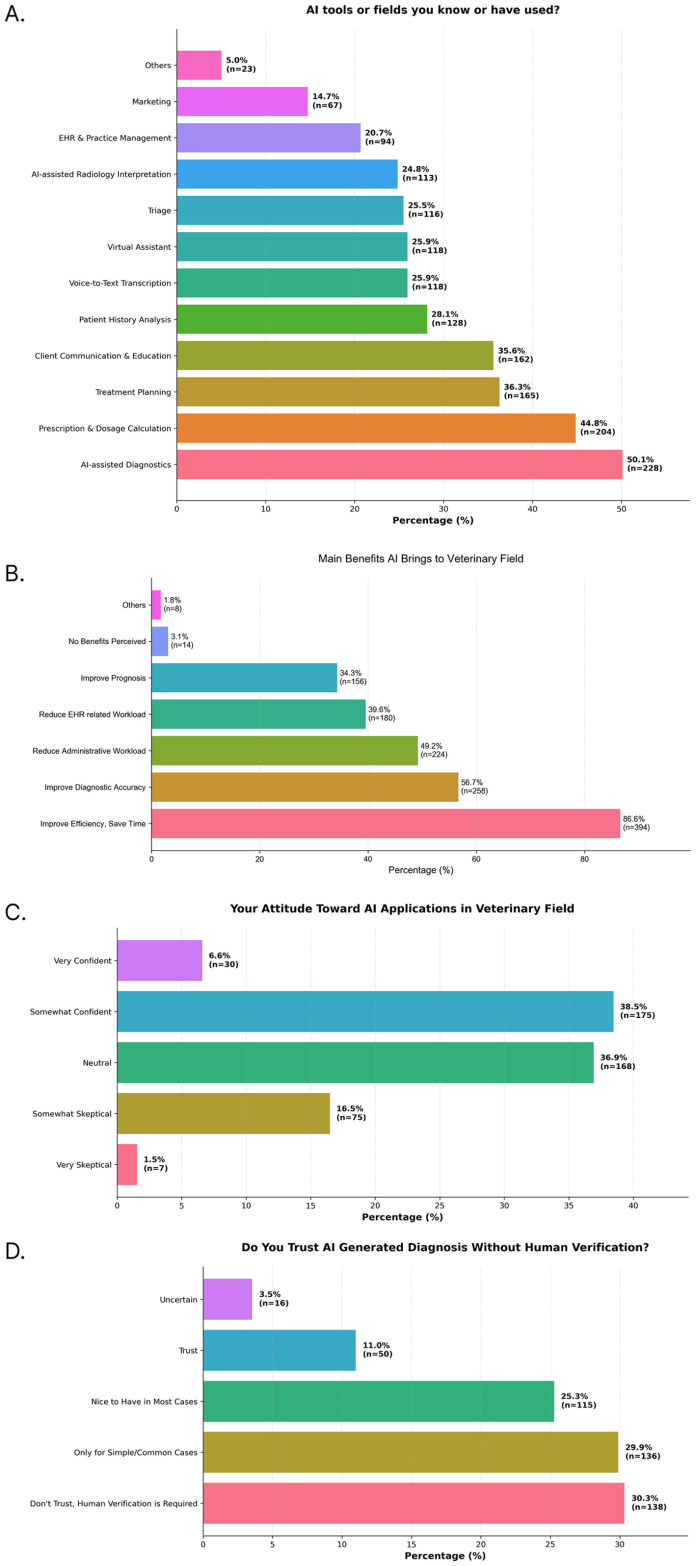
Survey results that highlight perceived benefits, attitudes, and trust in AI use in China. Bar charts show the distribution of responses to four survey questions: **(A)** AI tools or fields you know or have used? **(B)** Main benefits AI brings to veterinary field, **(C)** Your attitude toward ai applications in veterinary field, and **(D)** Do you trust AI diagnosis without human verification? Percentages for each category are displayed on the bars, along with the number of respondents (*n*) for that category.

Respondents primarily used AI for clinical decision-making tasks. The most cited application was AI-assisted disease treatment and diagnosis (50.1%, Figure 4A), followed by calculating and using prescription drugs (44.8%) and treatment planning (36.3%). More administrative functions, such as voice-to-text transcription (25.9%) and automation of electronic health records (20.7%), were cited less frequently. The most perceived benefit of AI was improving efficiency and saving time (86.6%, Figure 4B), followed by improving the accuracy of diagnosis (56.7%), reducing administrative workload (49.2%) and medical record related work (39.6%). While 45.1% of respondents (Figure 4C) expressed confidence in AI, 30.3% (Figure 4D) stated they do not trust unverified AI output for diagnosis.

### Barriers and drivers for future adoption in China

**Figure 5:**
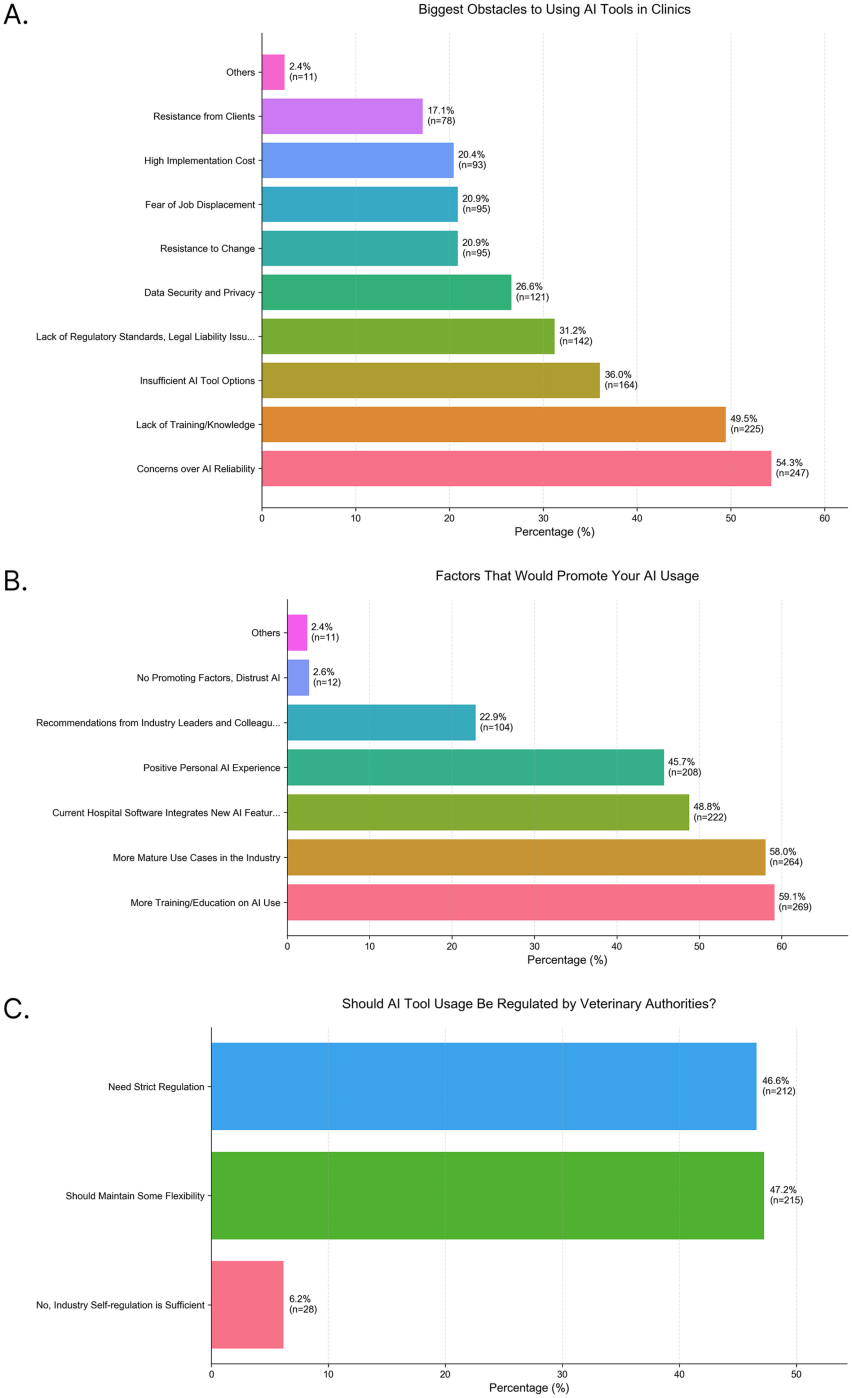
Survey results that highlight barriers to adoption and drivers for future use. Bar charts show the distribution of responses to three survey questions: **(A)** Biggest Obstacles to Using AI tools in Clinics, **(B)** Factors that would promote your AI usage, and **(C)** Should AI tool usage be regulated by veterinary authorities? Percentages for each category are displayed on the bars, along with the number of respondents (*n*) for that category.

The primary barrier to adoption was concern about AI reliability and accuracy (54.3%, Figure 5A). The second-most cited barrier was a lack of training and knowledge (49.5%). Consequently, the top-ranked factor that would promote usage was more training opportunities (59.1%, Figure 5B). A consensus of 93.8% of respondents (Figure 5C) believe AI use should be regulated by veterinary authorities, with opinion split between needing “strict regulation” (46.6%) and regulation that “should retain some flexibility” (47.2%).

### Descriptive comparison with north American data

When viewed alongside the previously published NA data, notable contrasting patterns in professional composition and adoption patterns are observed (Table 2). The Chinese cohort consisted of a higher proportion of veterinarians (81.5% vs. 24.3%) and reported a higher rate of AI adoption (71.0% vs. 39.2%). Application priorities also diverged. The Chinese cohort more frequently prioritized clinical tasks like diagnosis and disease detection (50.1% vs. 34.1%) and prescription calculation (44.8% vs. 17.6%). In contrast, the NA cohort more frequently reported using AI for administrative tasks like record-keeping (39.0% vs. 20.7%) and imaging and radiology (39.0% vs. 24.8%). Concerns about reliability and accuracy were the top barrier in both groups (NA: 70.3%, China: 54.3%). NA professionals rated data security and privacy (53.9% vs. 26.6% in China), cost (42.6% vs. 20.4% in China), and fear of job displacement (36.1% vs. 20.9% in China) as more prominent barriers than their Chinese counterparts.

**Table 2:** Descriptive comparison of key metrics between North American (2024) and Chinese (2025) veterinary professionals.

Comparison	North America (*n* = 3,968)	China (*n* = 455)
Composition		
Veterinarian	24.3%	81.5%
Tech/assistant	37.7%	5.9%
Vet student	13.6%	2.9%
Manager/exec	11.0%	4.0%
Receptionist	6.7%	1.3%
Other	6.6%	4.4%
Adoption and familiarity		
AI adoption rate	39.2%	71.0%
AI familiarity (Very/somewhat)	83.8%	44.6%
Primary applications		
Diagnosis and disease detection	34.1%	50.1%
Drug dosages and prescription	17.6%	44.8%
Treatment planning	31.1%	36.3%
Imaging and radiology	39.0%	24.8%
Record-keeping and admin tasks	39.0%	20.7%
Voice-to-text transcription	36.9%	25.9%
Client communication and education	31.7%	35.6%
Barriers		
Reliability and accuracy	70.3%	54.3%
Data security and privacy	53.9%	26.6%
Cost of implementation	42.6%	20.4%
Lack of sufficient tool options	Unclear	36.0%
Lack of training and knowledge	42.9%	49.5%
Regulatory or legal issues	42.1%	31.2%
Fear of job displacement	36.1%	20.9%

Direct statistical comparison is not appropriate due to significant differences in cohort composition, sampling frames, timing, and methodology. Data should be interpreted as highlighting contrasting contextual trends, not as evidence of population-level differences.

## Discussion

This study provides the first exploratory analysis of AI adoption among veterinary professionals in China, revealing a distinct integration approach. The central finding is an “adoption paradox”: while adoption is naturally highest among those with high familiarity (88.2%), our data reveals that 44.6% of active users report low familiarity with the technology. The co-existence of high usage and low familiarity suggests that this market is undergoing rapid, bottom-up technological assimilation. It paints a portrait of a profession in the early, enthusiastic, yet cautious stages of adoption, where practice is rapidly outpacing formal proficiency and trust. This “adoption paradox” helps us understand the unique dynamics of AI integration in China’s veterinary sector (companion animal care in particular).

The high adoption rate observed in this study is propelled by a powerful confluence of user demographics and systemic needs. The first driver is the demographic profile of the sampled respondents. Our cohort was predominantly young, with 57.8% born in the 1990s, which may reflect a broader trend of a youthful veterinary workforce in China and positions them as digital natives who are naturally inclined to leverage technology for problem-solving. They proactively experiment with accessible, general-purpose tools like large LLMs (e.g., Deepseek, used by 67.5% of respondents) to streamline their clinical workflow. This is consistent with a “bottom-up” adoption model, driven by individual practitioners rather than institutional mandate. The second, and arguably more profound, driver is a systemic need rooted in the structure of the veterinary profession in China. In the United States and Canada, veterinary medicine is a postgraduate profession requiring a doctoral degree (Doctor of Veterinary Medicine), ensuring a high and consistent standard of foundational clinical expertise. The system is additionally supported by a robust and accessible network of board-certified specialists (17). Whereas the pathway to veterinary licensure in China typically begins with a bachelor’s or even an associate degree from a vocational school (18–20). This results in a larger, more demographically varied professional base whose members may have little initial clinical training and, crucially, more limited access to board-certified specialists. In this context, AI is not merely a convenient tool for saving time; it evolves into a necessary form of digital decision support. However, this reliance carries inherent risks. In a professional landscape with variable baseline training, there is a heightened danger of uncritical acceptance of AI outputs, often termed “automation bias. “It is critical to emphasize that AI serves as a “digital clinical ally” to augment decision-making, not a replacement for the critical appraisal required for diagnosis and treatment. The ultimate professional responsibility remains with the clinician to verify AI suggestions against clinical evidence.

This drive for clinical augmentation is clearly reflected in the high prioritization of core clinical applications—AI-assisted disease diagnosis (50.1%), prescription calculation (44.8%), and treatment planning (36.3%)—which reveals an “inside-out” strategy. Here, AI is being leveraged from the very core of clinical practice to address potential knowledge gaps, mitigate the unevenness of foundational training, and standardize the quality of care across a diverse pet care landscape. For many practitioners, an AI diagnostic assistant is a powerful ally offering accessible second opinions, yet one that requires vigilant oversight given the potential for hallucination or error.

If AI is so integral to practice, what then explains the pervasive low familiarity and trust reported by these same users? The answer lies in the nature of the tools being used and the resulting “interpretability gap.” The current wave of AI adoption in China is dominated by general-purpose LLMs. While highly capable, these models often function as “black boxes” (21, 22); they provide outputs without transparent, step-by-step reasoning that a clinician can easily follow and verify. This lack of explainability is the fundamental barrier to both familiarity and trust. A veterinarian can use an LLM to generate a list of differential diagnoses but cannot be certain of the underlying clinical logic and the information source, potentially missing a crucial, low-probability but high-stakes condition. This erodes confidence and perpetuates a state of low familiarity, in other words, practitioners are actively utilizing the tools in their workflows but do not feel they understand the underlying technology or its reliability boundaries. This lack of algorithmic transparency is reflected in the data as well. While 45.1% of respondents expressed general confidence in AI, a substantial 30.3% of respondents stated they do not trust unverified AI output for diagnosis. Although our study’s demographic homogeneity (predominantly early-career) precludes a granular statistical comparison of trust across experience levels, the opaque nature of these tools suggests a differential impact. For the experienced, it breeds caution; for the less trained, it risks being overlooked in favor of utility. This sentiment was reinforced by qualitative comments describing tools as “sometimes wrong” or “too basic.” Therefore, the high usage exists within a carefully bounded comfort zone. Practitioners are willing to deploy AI for tasks where the output is verifiable (e.g., dose calculation, where a formula can be checked) or for generating initial ideas that will be scrutinized. However, they are hesitant to cede authority to AI for complex, high-stakes clinical decisions. This gap between using AI as an auxiliary tool and relying on it as a facilitated diagnostic partner is the crux of the adoption paradox. The profession, especially the experienced ones, is caught between recognizing AI’s immense utility and being aware of its current limitations and risks.

When the patterns observed in China are compared with the established data from North America, it becomes evident that the trajectory of a global technology is shaped by local ecosystems. The comparison reveals two contrasting adoption narratives, each logically consistent with its own context. The North American model, as reported in the 2024 survey, can be characterized as a cautious, “outside-in,” and provider-driven approach. With a highly standardized, postgraduate-trained workforce and a robust network of specialists, the most pressing daily challenges are often operational workload and rising administrative costs. Consequently, the initial integration of AI has logically focused on administrative tasks like record-keeping (39.0%) and voice-to-text transcription (36.9%). This is a strategy that initially targets areas with less direct clinical liability, building a foundation of efficiency upon which more advanced clinical tools might later be integrated.

In contrast, the Chinese model is a need-driven, “inside-out,” and practitioner-led phenomenon. The different participant composition, 81.5% veterinarians in China versus 24.3% in NA, is a key explanatory variable. This clinician-heavy sample, operating within an ecosystem of varied training and limited specialist access, has pivoted directly to using AI for clinical augmentation. This divergence demonstrates that adoption priorities are a direct reflection of local workforce demographics, educational structures, market maturity, and underlying professional needs. The trajectory of AI is not a monolithic, one-size-fits-all global rollout but a mosaic of regional adaptations.

The findings of this study carry significant implications for the global veterinary community. The strong consensus (93.8%) among Chinese respondents calling for regulatory oversight, coupled with their anxieties about liability and skill degradation, signals an urgent need for action. Professional associations and regulatory bodies should proactively develop standards for AI tool validation, algorithmic transparency, and clear liability frameworks. The question of “who is responsible? The doctor or the AI?” must be answered to prevent a chilling effect on adoption or, conversely, to prevent over-reliance on unvalidated tools (23). For educators, the data underscore a universal need to evolve veterinary curricula (24, 25). Moving beyond basic computer literacy, the next generation of veterinarians must be equipped with skills in data science and the ability to critically appraise AI technologies. The inclusion of courses focused on digital health ethics and AI literacy is a vital step in this direction. For technology developers, they should be aware that a new generation of veterinary-specific AI tools is needed. The demand for more specialized tool options and the interpretability gap create a clear market opportunity for solutions that are explainable, validated on veterinary data, and seamlessly integrated into clinical workflows.

Looking forward, this study suggests that global AI adoption will continue along multiple, parallel tracks. In mature markets, integration will likely remain systematic and caution-led, while in emerging markets, practitioner-driven adoption could accelerate, making it possible to leapfrog certain stages of development but also carrying heightened risks that require vigilant management. Future research must employ longitudinal designs to track this evolution and qualitative methods to thoroughly explore the clinical reasoning and decision-making processes of veterinarians using AI. Furthermore, studies investigating client acceptance and the economic models of AI-assisted services will be critical to understanding its ultimate role and sustainability in veterinary practice.

This study has several limitations that must be considered when interpreting the results. First, the use of a convenience sampling method, despite its strengths in reaching a broad national sample, may introduce selection bias (26). The recruitment through major conference and professional association channels likely overrepresents professionals who are more engaged with continuing education and potentially more technologically adept, which may inflate the reported adoption rates and familiarity. The inability to calculate a precise response rate due to the broad and diffuse nature of the sampling frame further complicates the assessment of this bias and the generalizability of the findings to the entire veterinary population in China. Second, the potential for ambiguity in key survey questions, particularly Question 8 (“Which AI Tools or Fields Have You Known or Used?”), which conflates awareness with actual use, may lead to an overestimation of adoption for specific applications. Third, the survey instrument, while carefully adapted and translated, lacks formal psychometric validation. Neither the original North American benchmark survey nor our adapted version underwent reliability or factor analysis testing. The findings should therefore be interpreted as descriptive indicators of perception and practice rather than as measurements from a clinically validated scale. Finally, while the descriptive comparison with North American data provides valuable international context, it is constrained by fundamental differences in cohort composition, sampling timeframes, and methodology. The lack of access to the raw NA dataset precluded any multivariable analysis to adjust for confounding factors, meaning the observed differences should be interpreted as generating hypotheses about regional variation rather than confirming them.

Furthermore, while the demographic profile of our respondents (e.g., a high proportion of young, small-animal veterinarians) is consistent with reported trends in China’s urban veterinary sector (27, 28), the convenience sampling method may not fully capture the diversity of the national veterinary workforce, including those in rural areas or large animal practice. This limits the generalizability of our findings to the entire veterinary population in China.

In conclusion, despite its limitations, this study provides a snapshot of AI adoption in a global market. The integration of AI into veterinary medicine is a complex, socially embedded process. The adoption paradox in China, high use amid low familiarity, is an example of this complexity. By illuminating this unique pathway, our study moves the conversation beyond simple adoption metrics and into the richer territory of how and why technology is adopted in different contexts. For all stakeholders, from clinicians and educators to developers and regulators, success will depend on recognizing and responding to these distinct regional narratives. By doing so, the global veterinary community can responsibly guide the development of AI, ensuring it truly advances animal health and empowers, rather than replaces, the veterinary professionals dedicated to it.

## Data Availability

The original contributions presented in the study are included in the article/Supplementary material, further inquiries can be directed to the corresponding author.
