# Supplementary material for: The adoption paradox for veterinary professionals in China: high use of artificial intelligence despite low familiarity
Source: Front Vet Sci. 2026 Mar 11;13:1727001. doi: 10.3389/fvets.2026.1727001 (PMC13012951; doi:10.3389/fvets.2026.1727001)

**Supplemental material:**

**Full questionnaire - Original version (Chinese)**

1. 基本信息
2. 您的年龄？
   1. 00后
   2. 90后
   3. 80后
   4. 70后
   5. 60后
3. 您在兽医学领域的主要工作是？
   1. 兽医
   2. 兽医技师/助理/护士
   3. 兽医学生
   4. 诊所管理者
   5. 诊所前台
   6. 其他
4. 您从事兽医学领域的工作时长？
   1. < 1年
   2. 1-5年
   3. 6-10 年
   4. > 10年
5. 您所在的机构类型？
   1. 宠物医院或诊所
   2. 大动物诊疗机构
   3. 异宠诊疗机构
   4. 疫苗/宠物食品 /保健品等公司
   5. 高校或科研院所
   6. 政府
6. AI在个人和医疗上的使用
7. 您对AI在兽医学中的应用有多熟悉，如ChatGPT, Deepseek？
   1. 非常熟悉
   2. 比较熟悉
   3. 不太熟悉
   4. 完全不熟悉
8. 您是否在兽医实践中使用过AI工具？
   1. 是
   2. 否
9. 您在兽医实践中使用AI工具的频率？
   1. 每天
   2. 每周
   3. 每月
   4. 很少使用，或从未用过
10. 以下涉及AI的工具或领域您了解或使用过哪些？(可多选)
    1. 放射影像分析（如AI辅助X光片解读）
    2. 电子健康记录（EHR）以及医院管理工作的自动化
    3. 语音对文字的自动转化
    4. AI辅助的疾病诊疗和诊断
    5. 宠主的沟通和教育
    6. 对患者病史的分析
    7. 治疗方案的设计
    8. 临床上的分诊
    9. 市场营销和推广
    10. 处方药的计算和使用
    11. 虚拟多功能兽医助手
    12. 其他
11. **您是否在个人的生活中使用过**AI工具？(可多选)
    1. Deepseek
    2. 豆包
    3. Kimi Chat
    4. 秘塔AI
    5. ChatGLM/智谱清言
    6. 宠智灵科技
    7. 谛宝扁鹊
    8. 微宠医
    9. 贝塔猫
    10. ChatGPT, Gemini, Claude, Llama, Grok等大语言模型
    11. iCHONG AI
    12. 其他
    13. 无使用经验
12. 您对AI工具的使用体验
    1. **试过，但不适合我/不满意效果**
    2. **尝试了一些，准备继续探索**
    3. **在日常生活中经常使用**
13. **对**AI应用的态度和看法
14. **您对AI在兽医领域的应用持什么态度？**
    1. **非常怀疑**
    2. **有一定质疑**
    3. **中立**
    4. **有一定信心**
    5. **非常有信心**
15. 您认为AI为兽医领域带来的主要好处是？(可多选)
    1. 提高效率，节省时间
    2. 减少病例输入及管理的负担
    3. 减少行政相关工作
    4. 提高诊疗的准确率
    5. 改善患者的预后情况
    6. 不认为有任何好处
    7. 其他
16. 以下哪个领域您认为会最大程度地享受AI带来的红利？(可多选)
    1. 宠主的沟通和教育
    2. 患者病例的填写
    3. 诊疗前的初诊和分诊
    4. 后续的返诊
    5. 治疗方案的安排
    6. 远程医疗
    7. 诊断检查，如血检、病理切片分析等
    8. 鉴别诊断
17. 您认为在诊所中使用AI工具最大的障碍是什么？(可多选)
    1. 使用成本高
    2. 缺乏培训/知识
    3. 对变革/变化的抵触
    4. 对AI可靠性的担忧
    5. AI工具可选性不足
    6. 缺乏监管标准，涉及的法律责任等
    7. 担心被AI取代，工作机会流失
    8. 内部数据的安全性没有保障
    9. 宠主/畜主的抵触
    10. 其他
18. 您认为以下哪些因素会促进您对AI的使用？(可多选)
    1. 行业出现较多比较成熟的使用案例
    2. 更多的培训机会去增加对AI使用的了解
    3. 个人对AI使用的正面经历
    4. 诊所当下使用的软件整合了新的AI功能
    5. 行业中其他领军人以及同事的推荐
    6. 没有可以促进的因素，不信任AI
    7. 其他
19. 您认为对兽医从业者对AI方面的培训有多重要？
    1. 非常重要
    2. 比较重要
    3. 不重要
    4. 不确定
20. 您是否信任未经人工核查的AI初步诊断？
    1. 信任
    2. 多数情况表现良好
    3. 仅信任简单或常见病例
    4. 不信任，AI仅仅是辅助工具，必须进行人工复核
    5. 不确定
21. 您认为畜主/宠主会接受AI辅助的诊断/治疗么？
    1. 大多数接受
    2. 部分接受，部分犹豫
    3. 大部分倾向纯人工诊疗
    4. 不确定
22. 您认为结合AI工具会提高诊所的竞争力么？
    1. 是
    2. 否
23. 您在短期内会考虑进一步扩大诊所内AI的使用频率和范围么？
    1. 不考虑
    2. 不确定
    3. 会考虑，但是会谨慎使用
24. 您认为AI工具的使用是否应由兽医监管机构统一规范？
    1. 需要严格监管
    2. 应保留一定灵活性
    3. 否，行业自调即可
25. 开放性问题
26. 请描述您在兽医实践中使用AI的积极或者消极的经历。
27. 您对AI在动物医疗的伦理问题有何担忧。
28. 您希望未来的AI兽医工具具备哪些功能。
29. 您认为未来十年AI将如何改变兽医的角色。
30. 关于AI在兽医学中的应用，您还有其他意见或建议么？

**Full questionnaire – Translated version (English)**

I. Demographic Information

1. Your Age:

1. Post-00s (Born after 2000)
2. Post-90s (Born after 1990)
3. Post-80s (Born after 1980)
4. Post-70s (Born after 1970)
5. Post-60s (Born after 1960)

2. Your Role in Veterinary Field

1. Veterinarian
2. Veterinary Technician/Assistant/Nurse
3. Veterinary Student
4. Clinic Manager
5. Clinic Receptionist
6. Other

3. Your Work Experience in Veterinary Field

1. < 1 year
2. 1-5 years
3. 6-10 years
4. > 10 years

4. Type of Institution You Work At

1. Pet Hospital/Clinic
2. Large Animal Clinic
3. Exotic Pet Clinic
4. Vaccine/Pet Food/Health Product Company
5. University/Research Institute
6. Government

II. AI Use in Personal and Medical Applications

5. Your Familiarity with AI Applications in Veterinary Medicine, such as ChatGPT, Deepseek?

1. Very Familiar
2. Somewhat Familiar
3. Not Very Familiar
4. Completely Unfamiliar

6. Have You Used AI Tools in Veterinary Practice?

1. Yes
2. No

7. Frequency of AI Tool Usage in Veterinary Practice

1. Daily
2. Weekly
3. Monthly
4. Rarely use

8. Which AI Tools or Fields Have You Known or Used? (Multiple Choice)

1. AI-assisted Radiology Interpretation
2. EHR & Practice Management
3. Voice-to-Text Transcription
4. AI-assisted Diagnostics
5. Pet Owner Communication and Education
6. Patient History Analysis
7. Treatment Planning
8. Triage
9. Marketing
10. Prescription & Dosage Calculation
11. Virtual Assistant
12. Other

9. Have you used AI tools in your personal life? (Multiple Choice)

1. Deepseek
2. DouBao
3. Kimi Chat
4. Mita AI
5. ChatGLM/Zhipu AI
6. Chongzhiling
7. Dibaobianque
8. Mini Vet
9. Beta Cat
10. ChatGPT, Gemini, Claude, Llama, Grok and other LLMs
11. iCHONG AI
12. Other
13. Never Used

10. Your Experience with AI Tools

1. Tried, but unsatisfied with results
2. Tried some, plan to continue
3. Positive experience, frequently used in daily life

III. Attitudes and Perspectives on AI Applications

11. Your Attitude Toward AI Applications in Veterinary Field

1. Very Skeptical
2. Somewhat Skeptical
3. Neutral
4. Somewhat Confident
5. Very Confident

12. Main Benefits AI Brings to Veterinary Field (Multiple Choice)

1. Improve Efficiency, Save Time
2. Reduce EHR related Workload
3. Reduce Administrative Workload
4. Improve Diagnostic Accuracy
5. Improve Prognosis
6. No Benefits Perceived
7. Others

13. Which Field Will Benefit Most from AI? (Multiple Choice)

1. Client Communication and Education
2. Medical Record Writing
3. Clinical Triage
4. Follow-up Visits
5. Treatment Plan
6. Telemedicine
7. Diagnostics, such as blood tests, pathological slide analysis, etc.
8. Differential Diagnosis

14. Biggest Obstacles to Using AI Tools in Clinics (Multiple Choice)

1. High Implementation Cost
2. Lack of Training/Knowledge
3. Resistance to Change
4. Concerns about AI Reliability
5. Insufficient AI Tool Options
6. Lack of Regulatory Standards, Legal Liability Issues
7. Fear of Job Displacement
8. Data Security and Privacy
9. Resistance from Clients
10. Others

15. Factors That Would Promote Your AI Usage (Multiple Choice)

1. More Mature Use Cases in the Industry
2. More Training/Education on AI Use
3. Positive Personal AI Experience
4. Current Hospital Software Integrates New AI Features
5. Recommendations from Industry Leaders and Colleagues
6. No Promoting Factors, Distrust AI
7. Others

16. Importance of AI Training for Veterinary Practitioners

1. Very Important
2. Somewhat Important
3. Not Important
4. Uncertain

17. Do You Trust AI Preliminary Diagnosis Without Human Verification?

1. Trust
2. Good Performance in Most Cases
3. Only Trust for Simple/Common Cases
4. Don't Trust, AI is Only Assistive, Human Verification is Required
5. Uncertain

18. Do You Think Pet Owners Will Accept AI-Assisted Diagnosis/Treatment?

1. Most Accept
2. Some Accept, Some Hesitate
3. Most Prefer Veterinarians
4. Uncertain

19. Can AI Tools Improve Medical Institution Competitiveness?

1. Yes
2. No

20. Will You Consider Expanding AI Usage in Your Institution in the Short Term?

1. Not Considering
2. Uncertain
3. Will Consider, But Use Cautiously

21. Should AI Tool Usage Be Regulated by Veterinary Authorities?

1. Need Strict Regulation
2. Should Maintain Some Flexibility
3. No, Industry Self-regulation is Sufficient

IV. Open-ended Questions

22. Please describe your positive or negative experiences using AI in veterinary practice.

23. What are your concerns about the ethical issues of AI in animal healthcare?

24. What features do you hope future AI veterinary tools will have?

25. How do you think AI will change the role of veterinarians in the next ten years?

26.Do you have any other opinions or suggestions regarding the application of AI in veterinary medicine?

**Supplementary Figures: Visualizations of Response Distributions (Questions 1–21)**


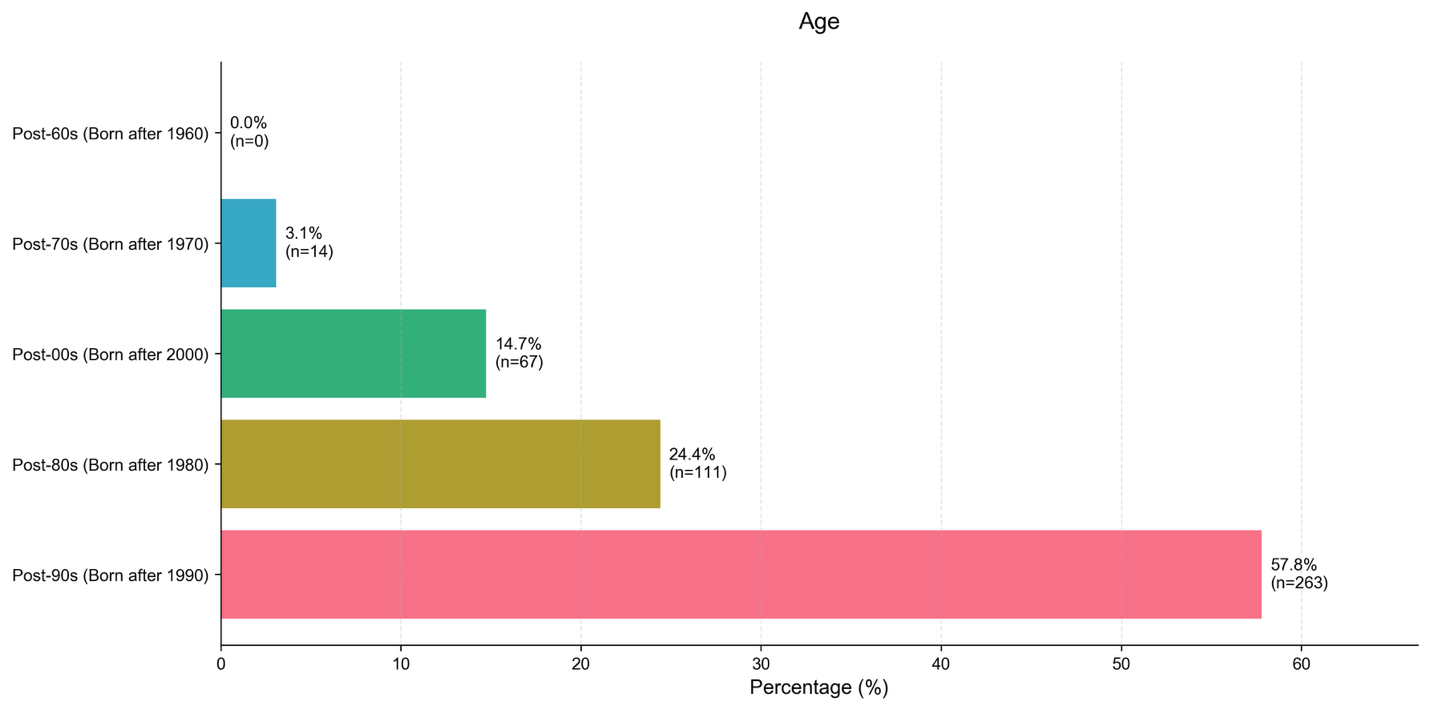

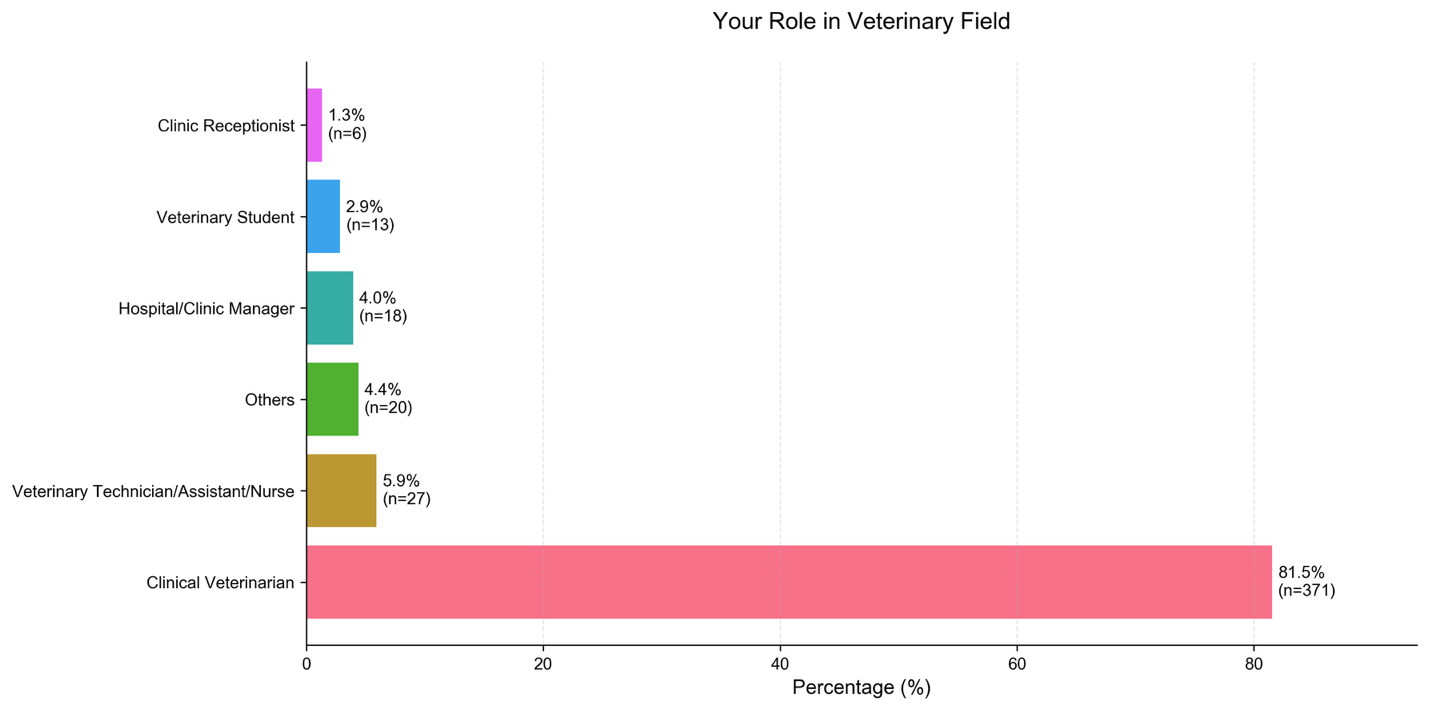

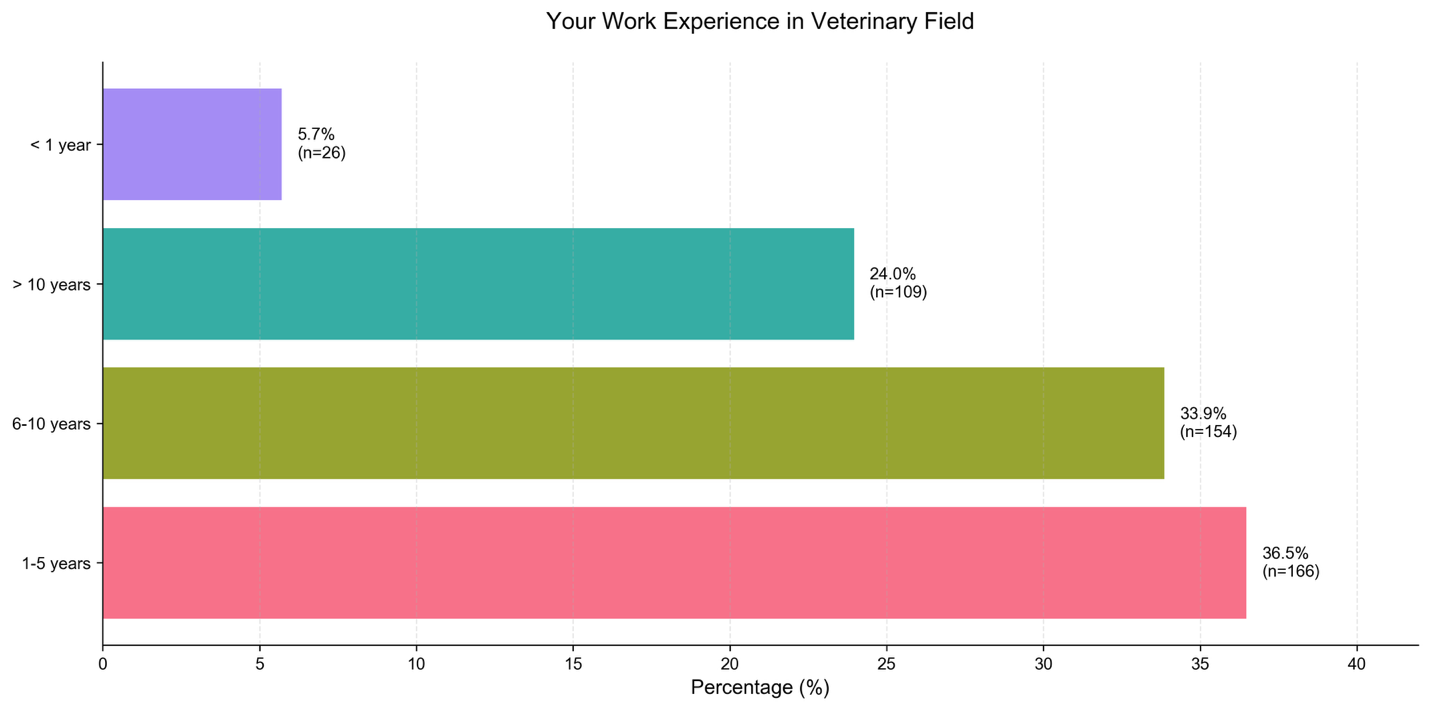

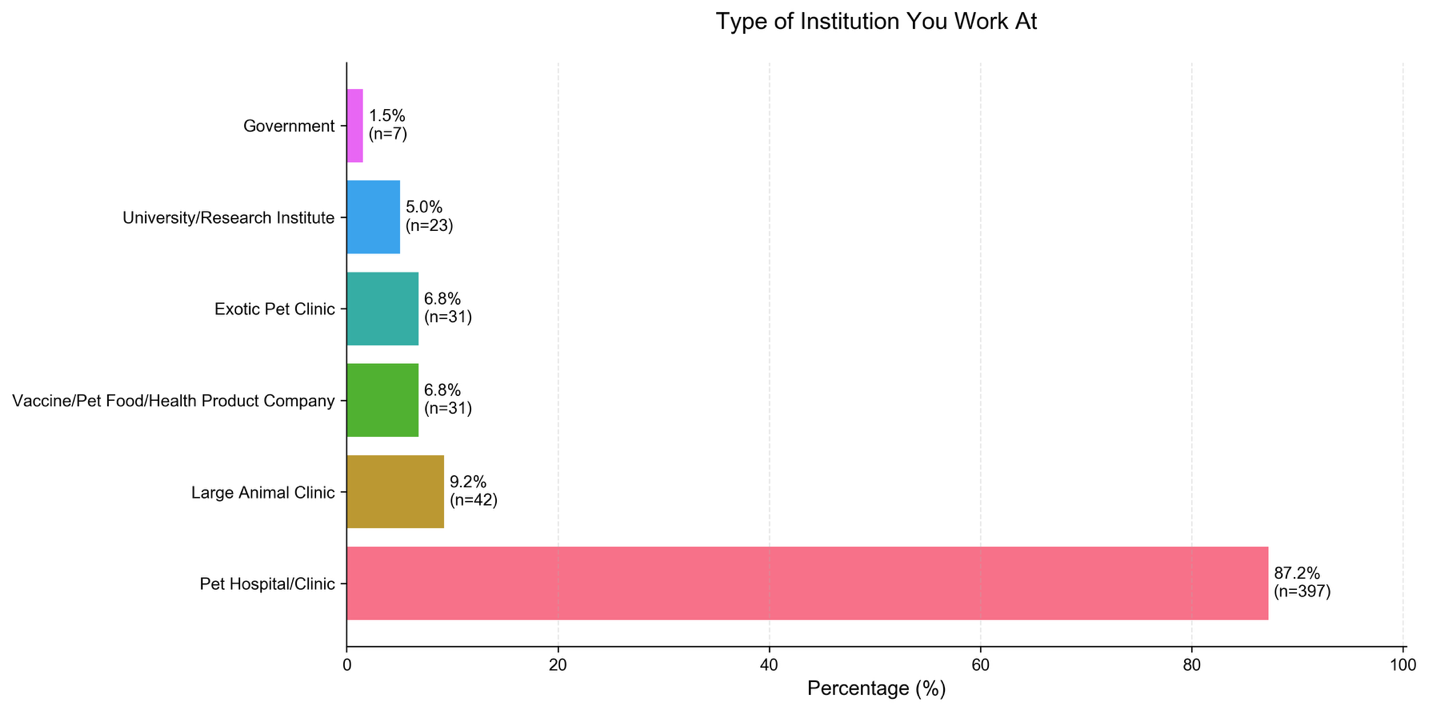

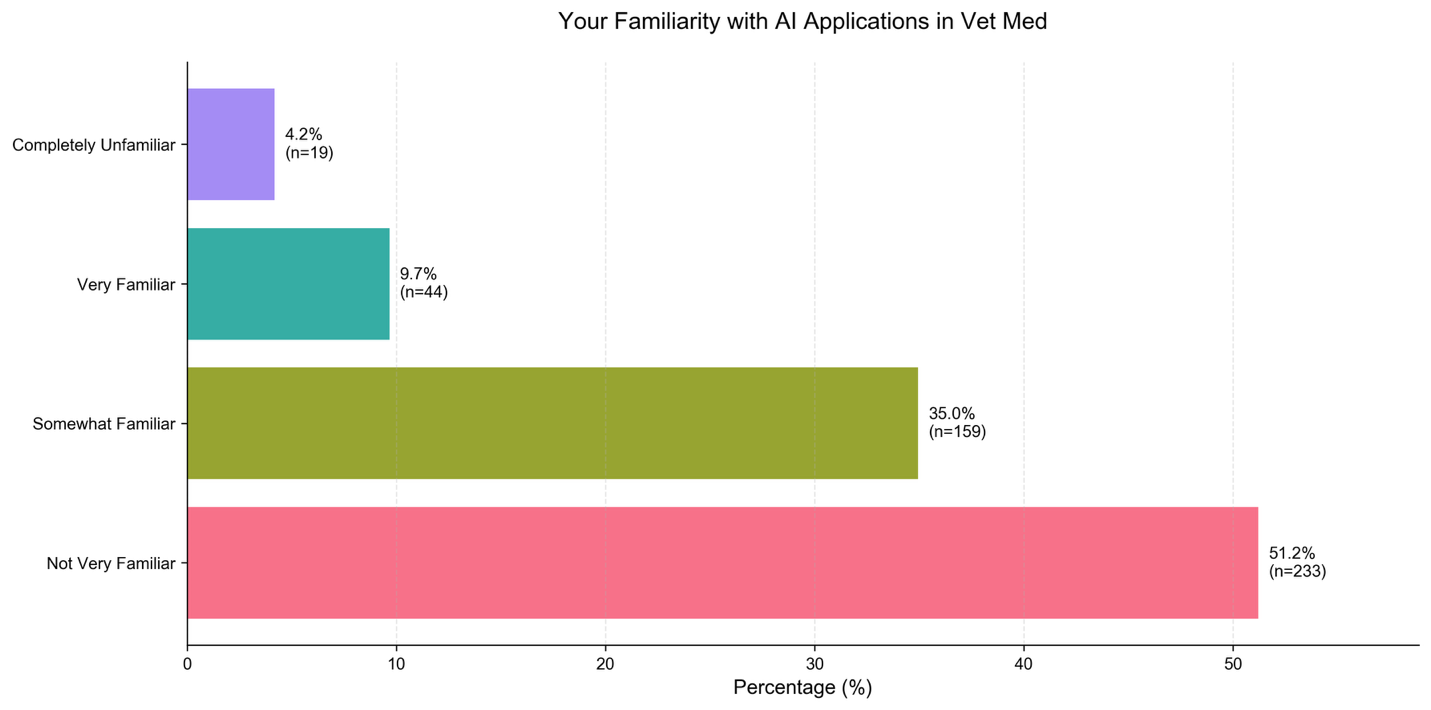

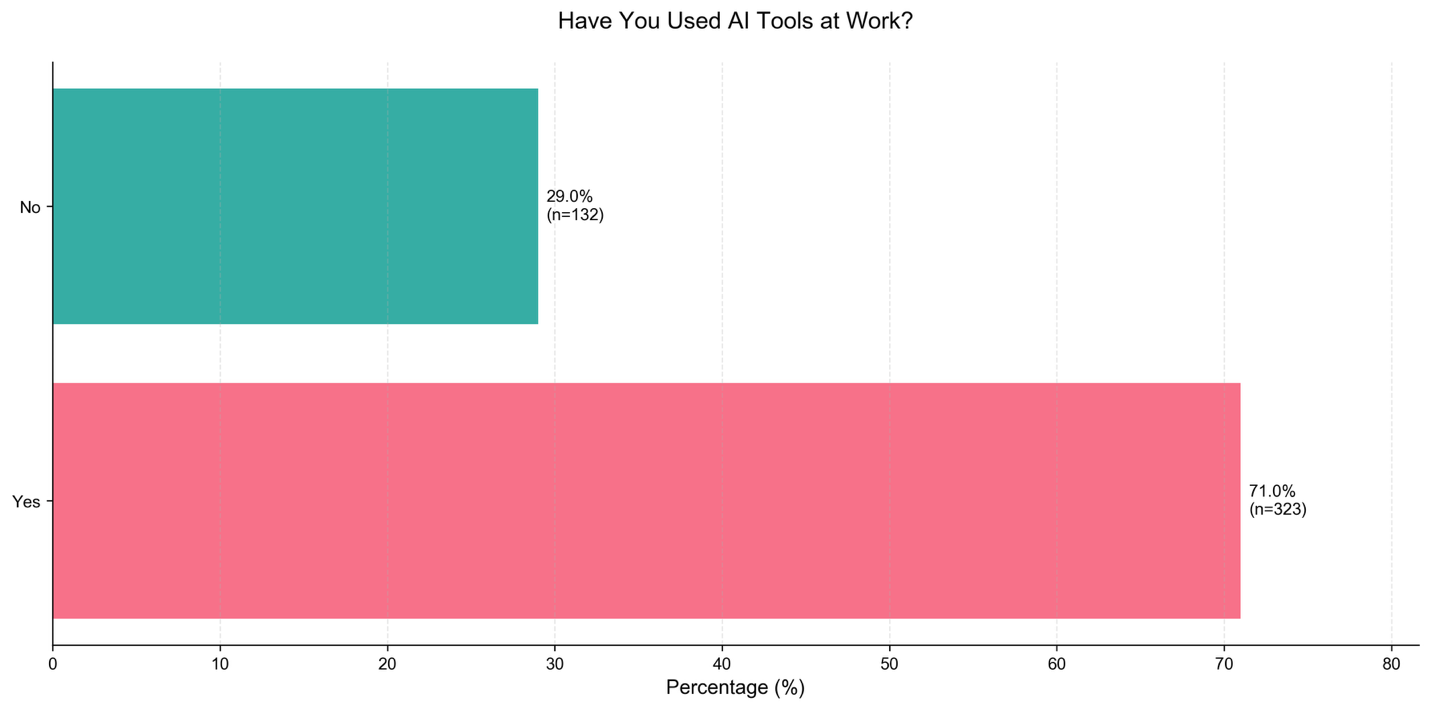

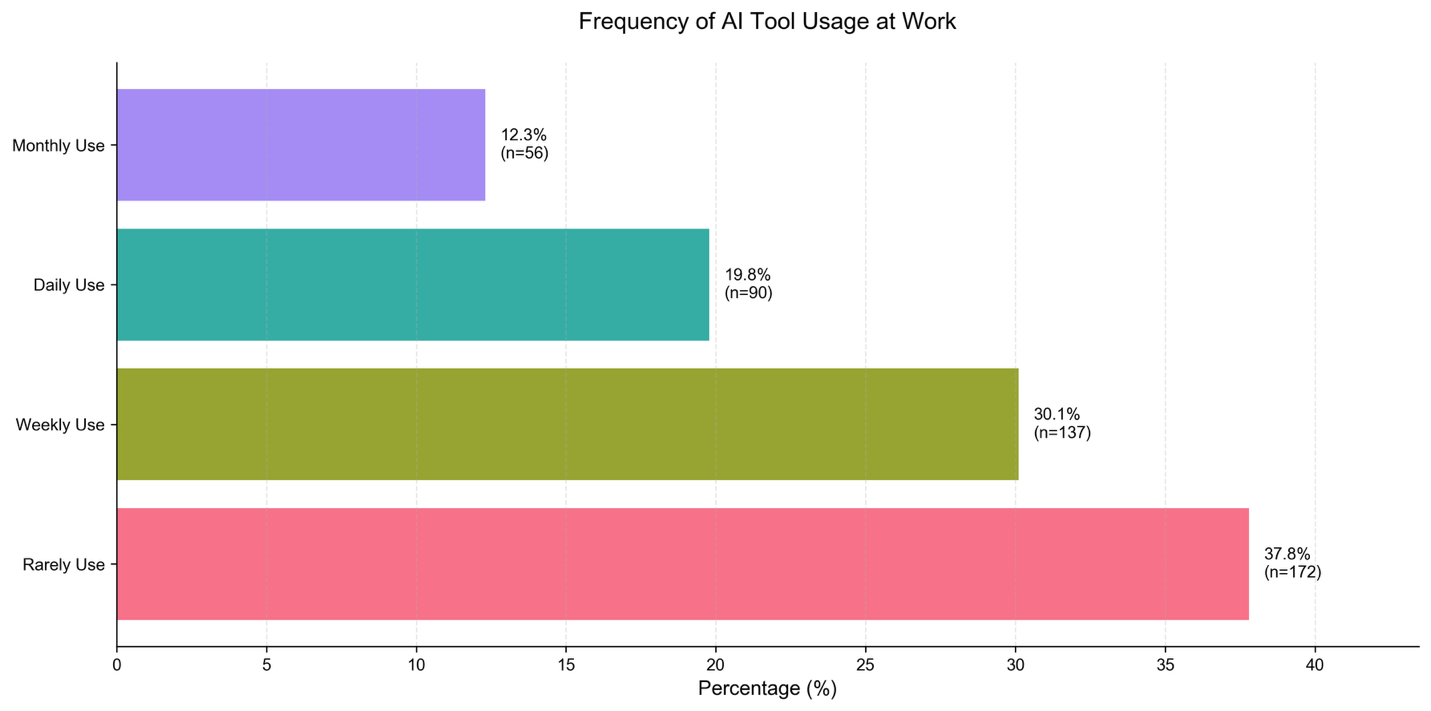

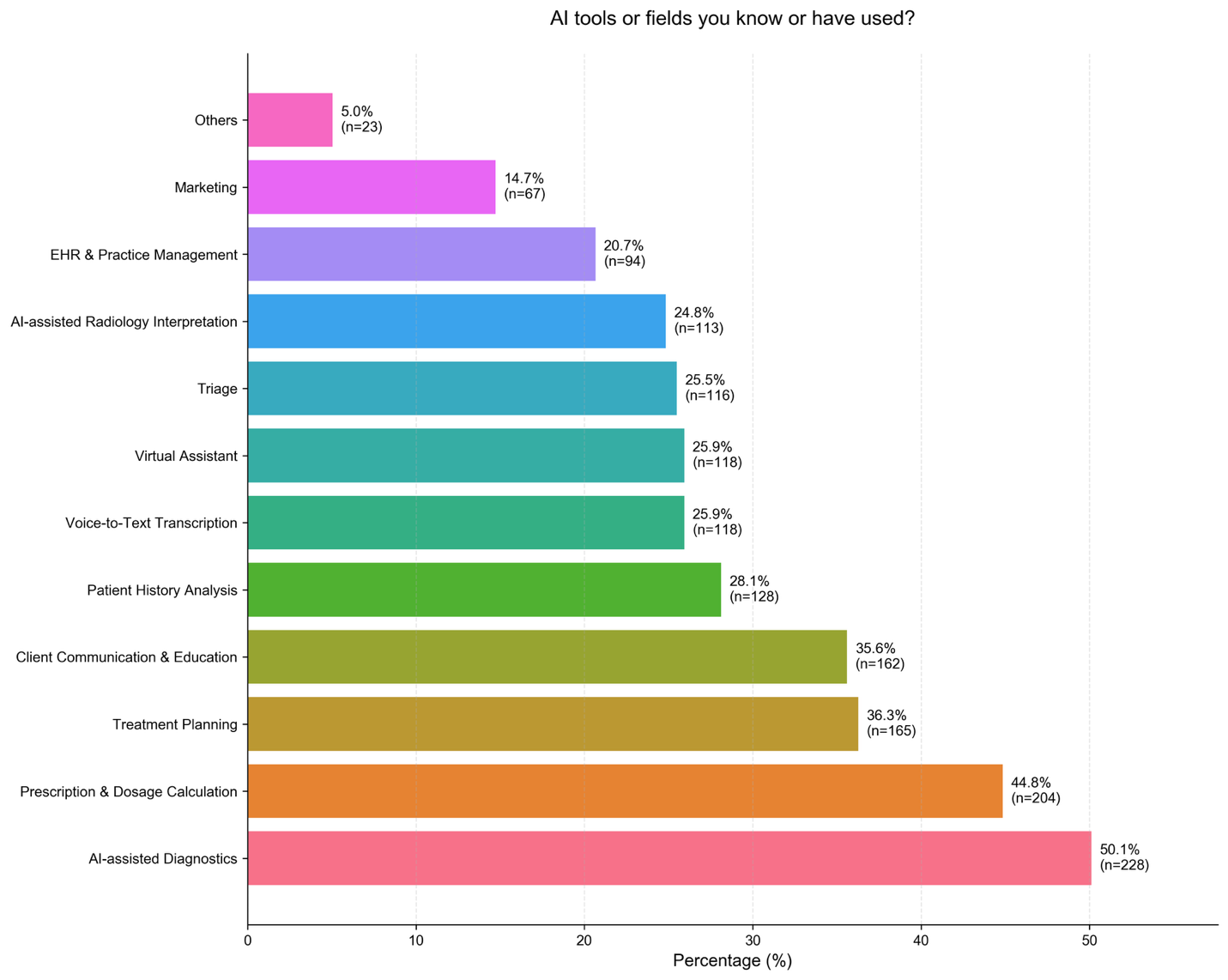

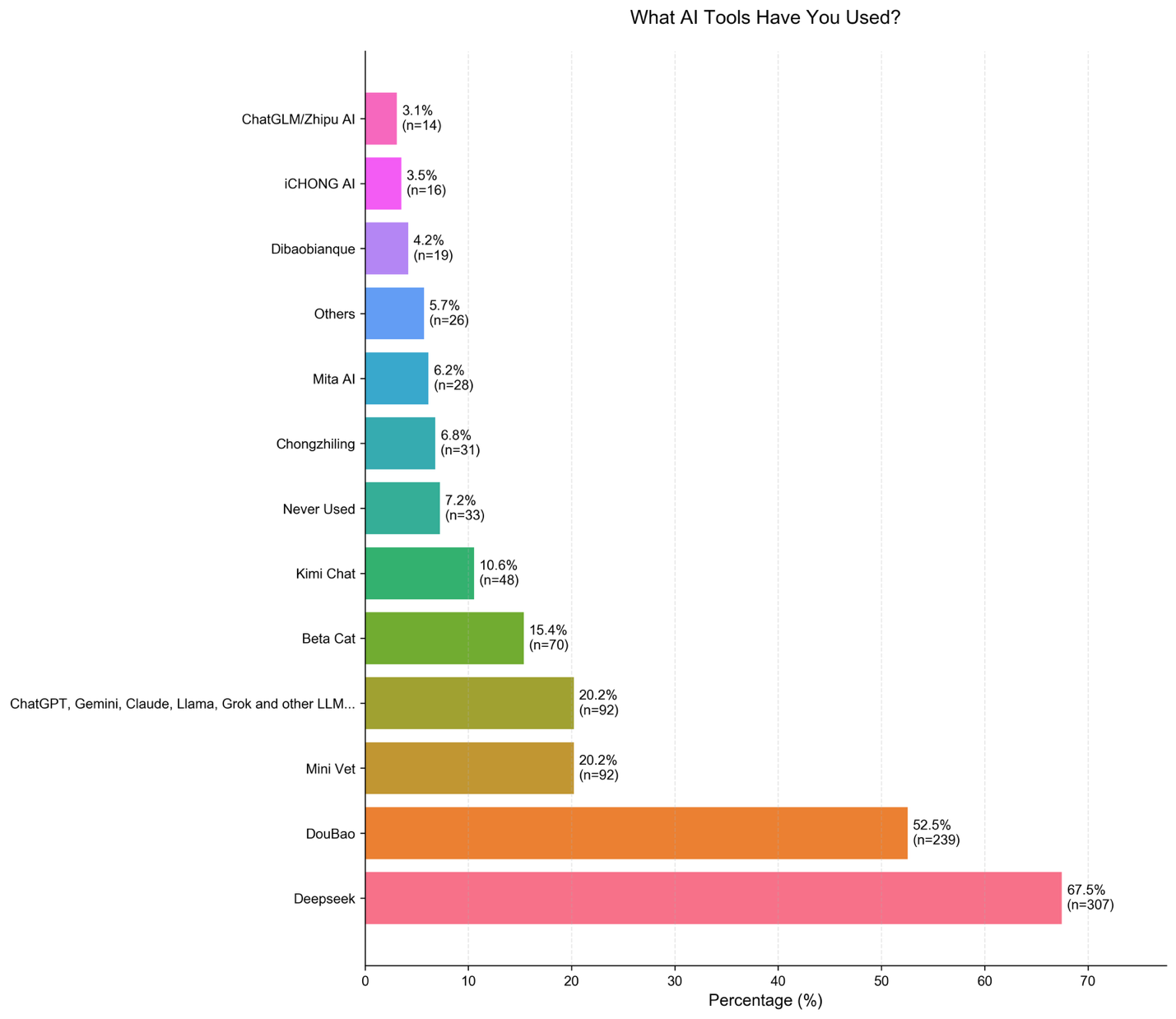

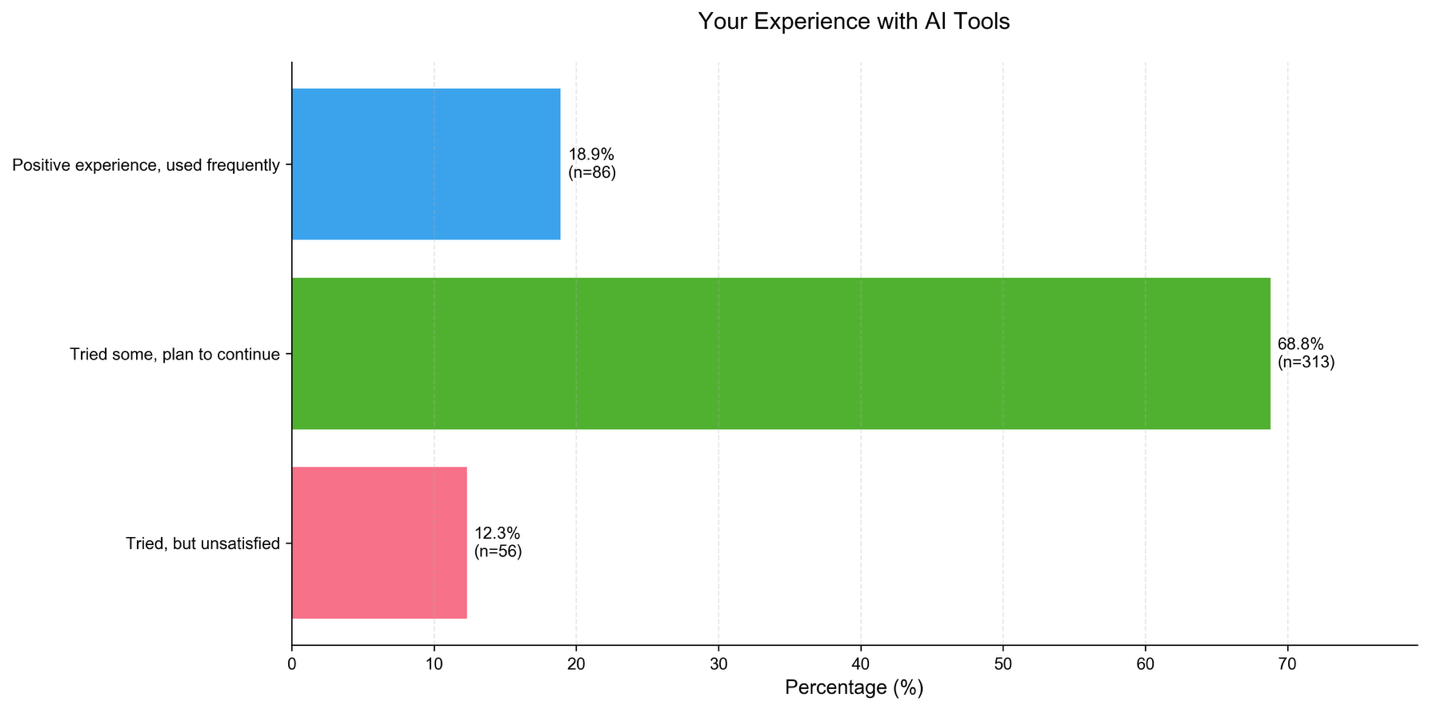

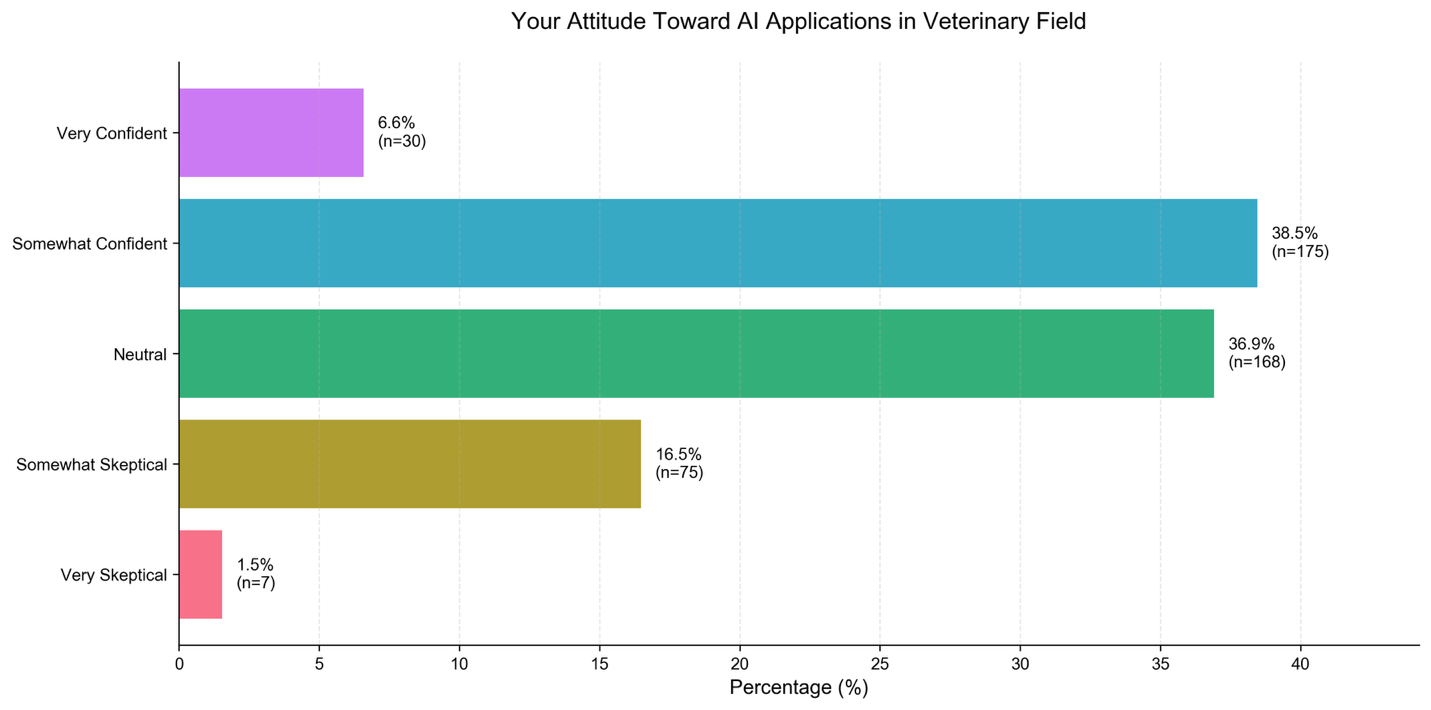

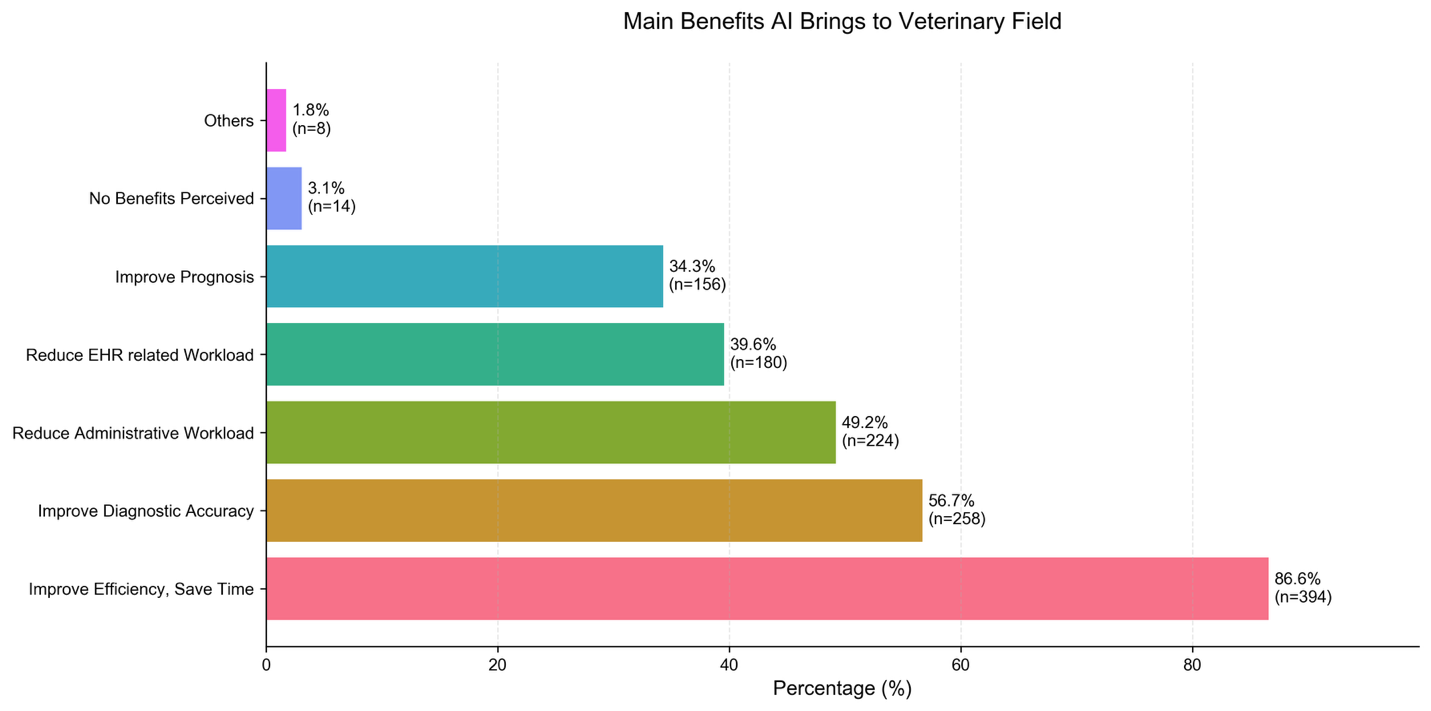

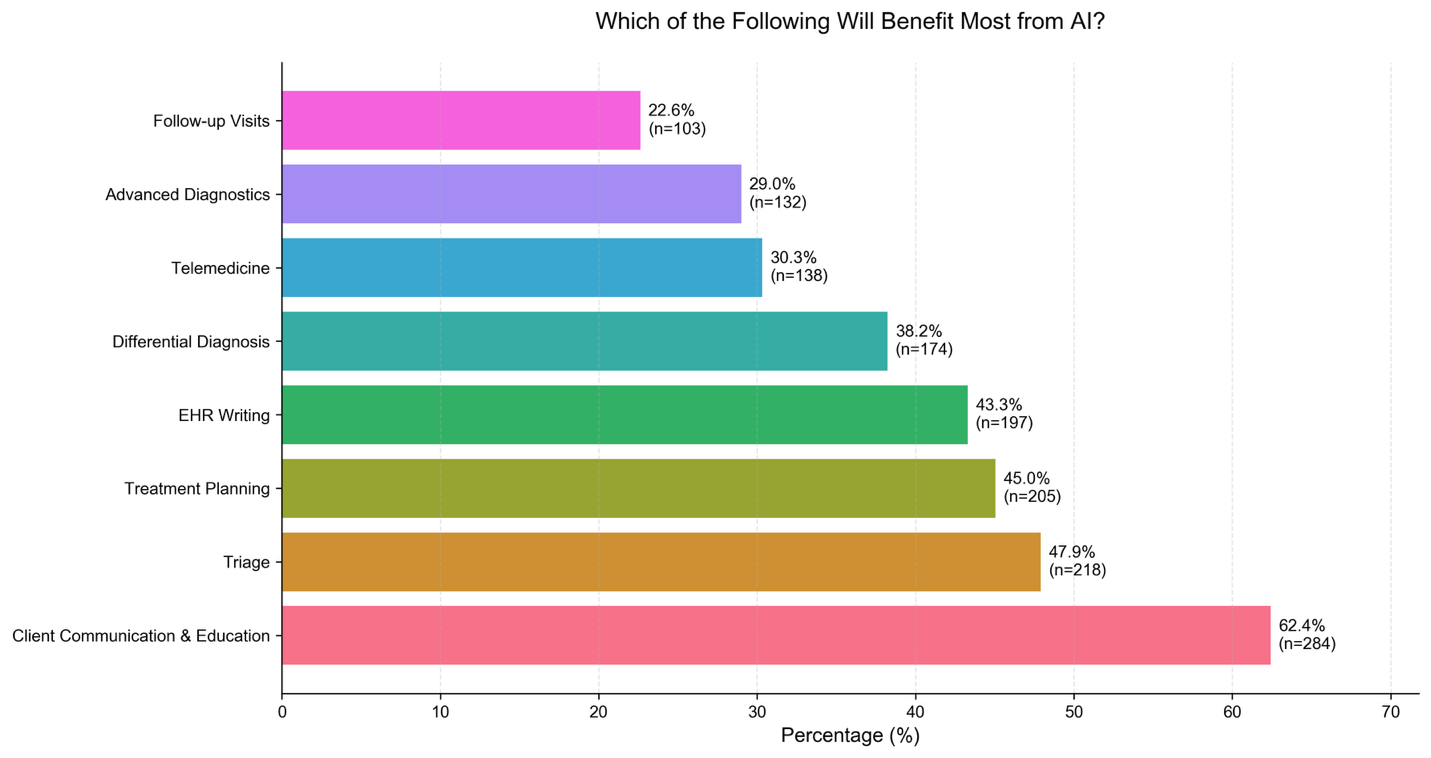

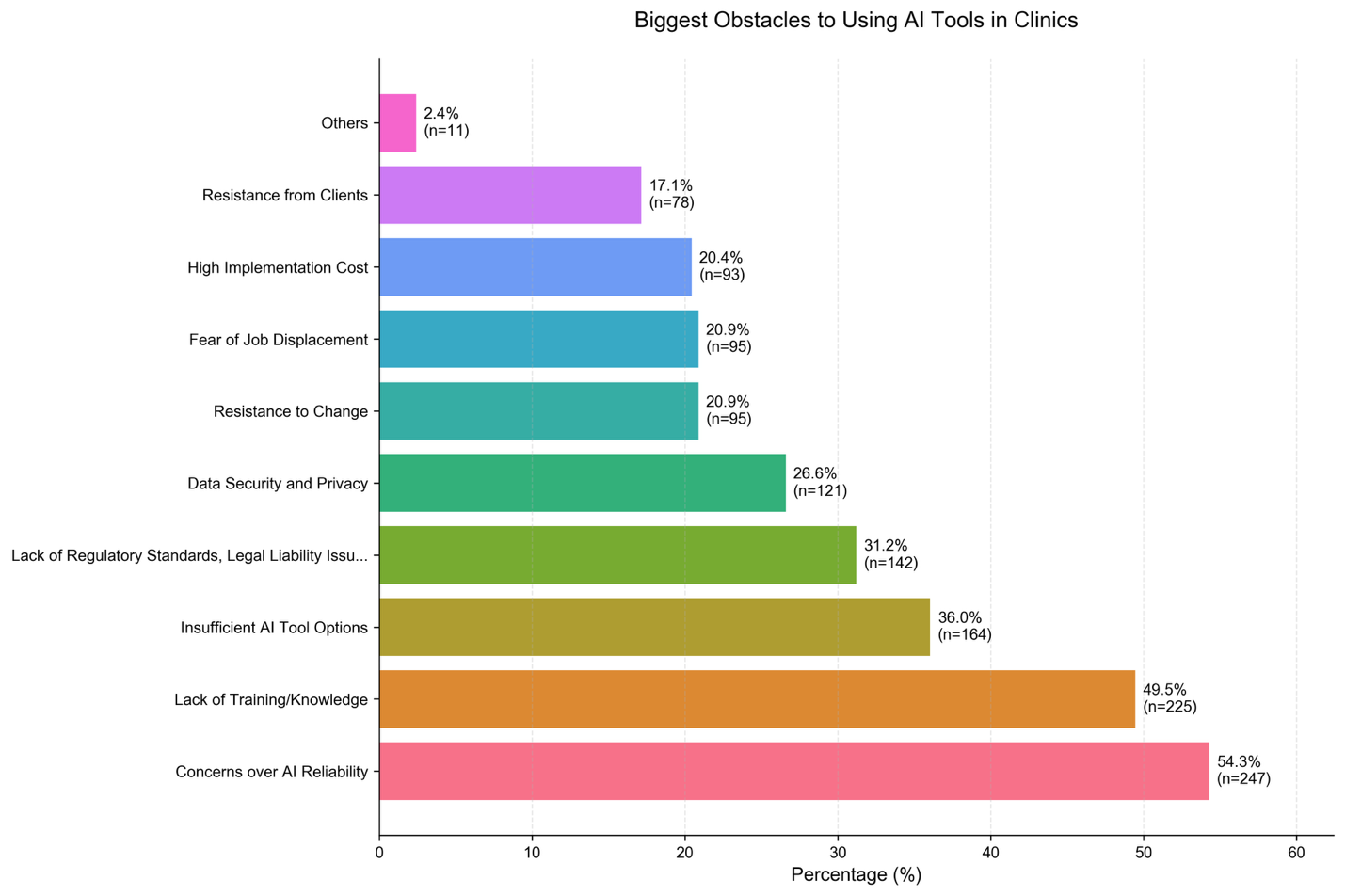

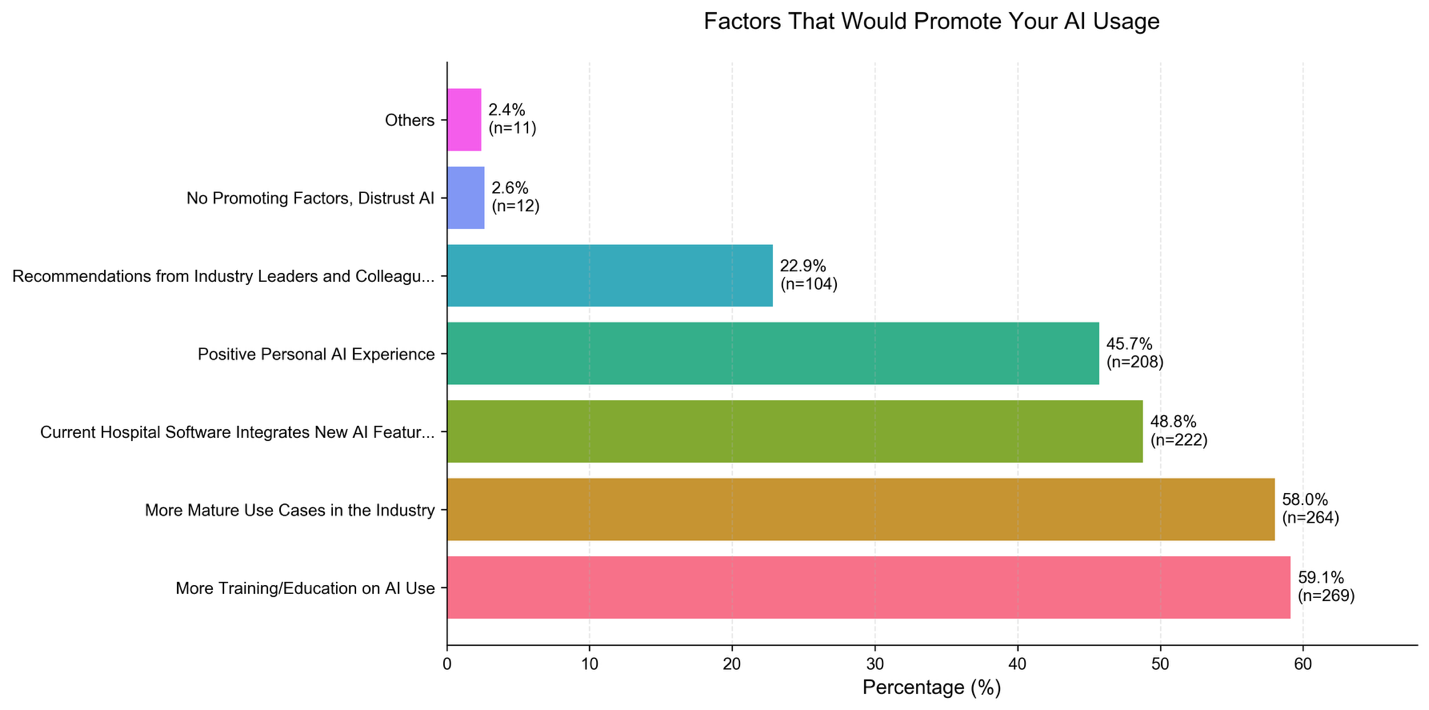

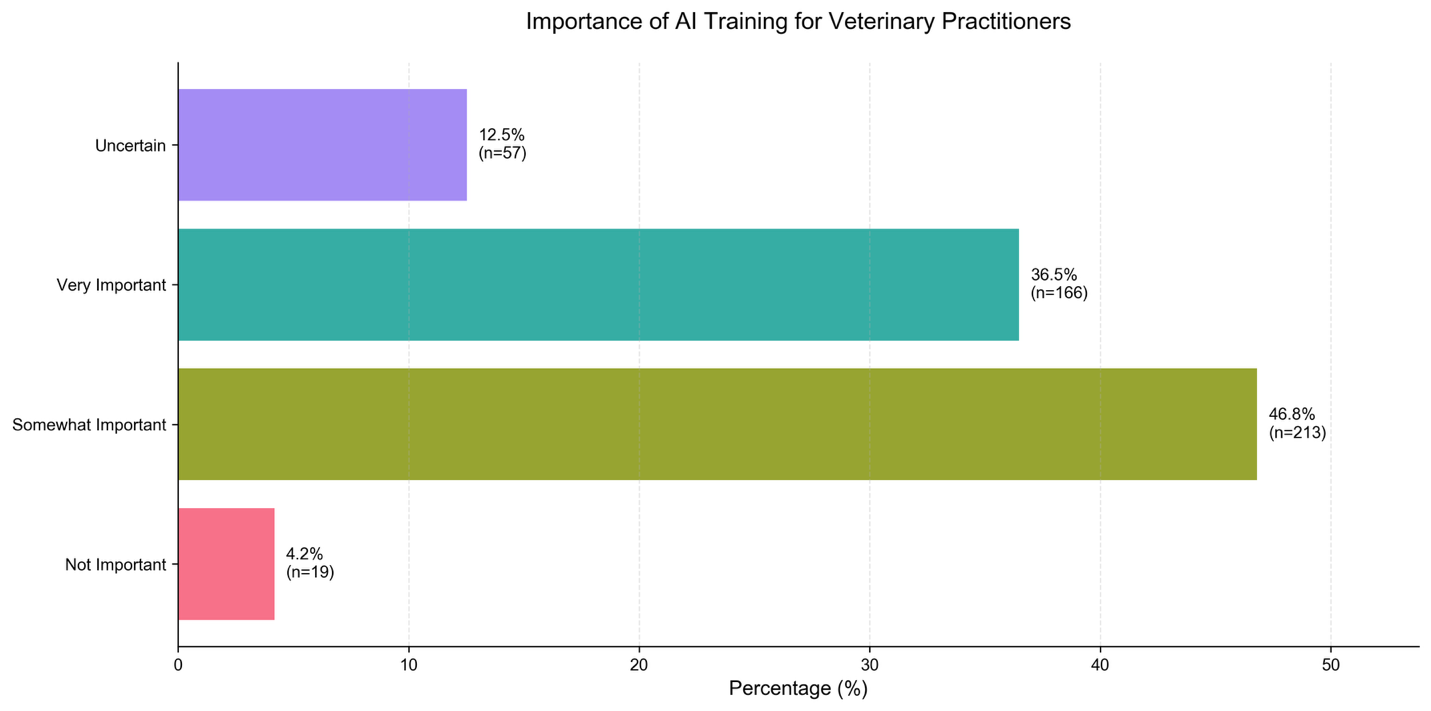

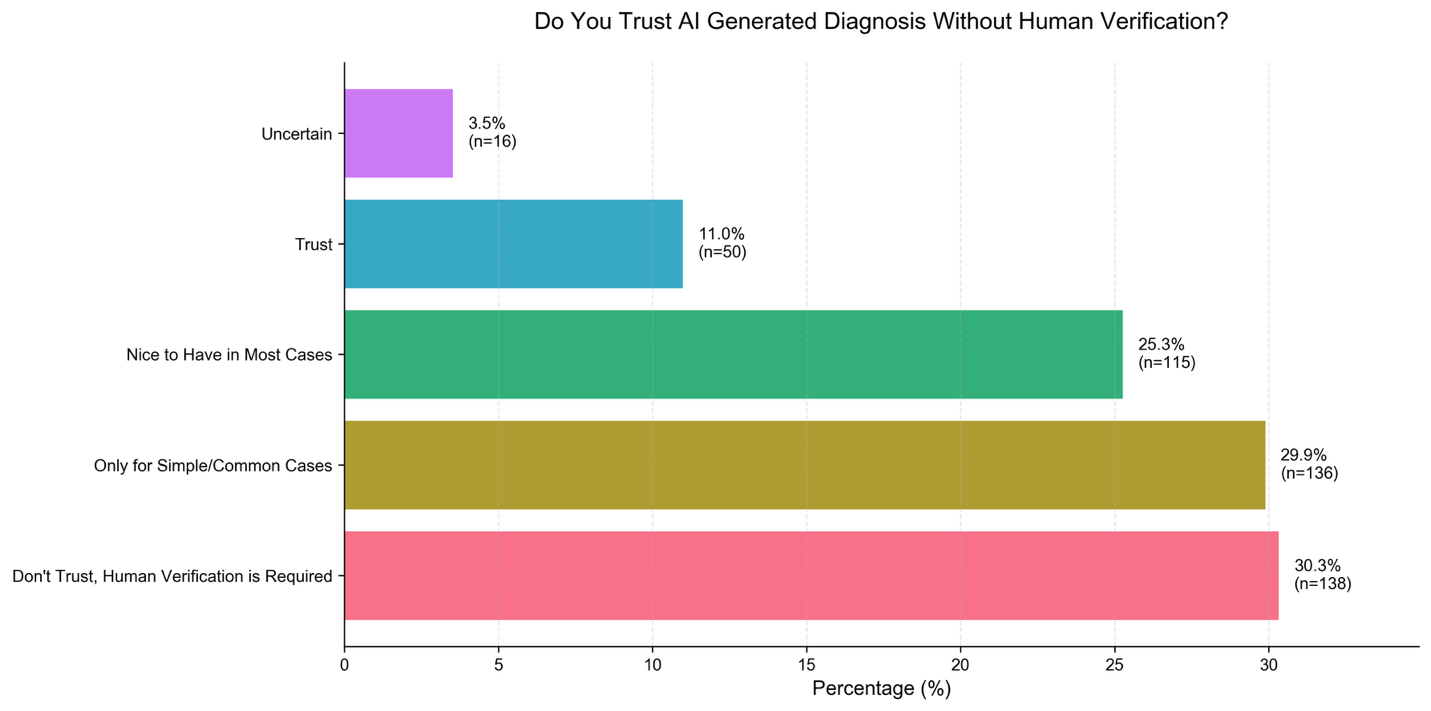

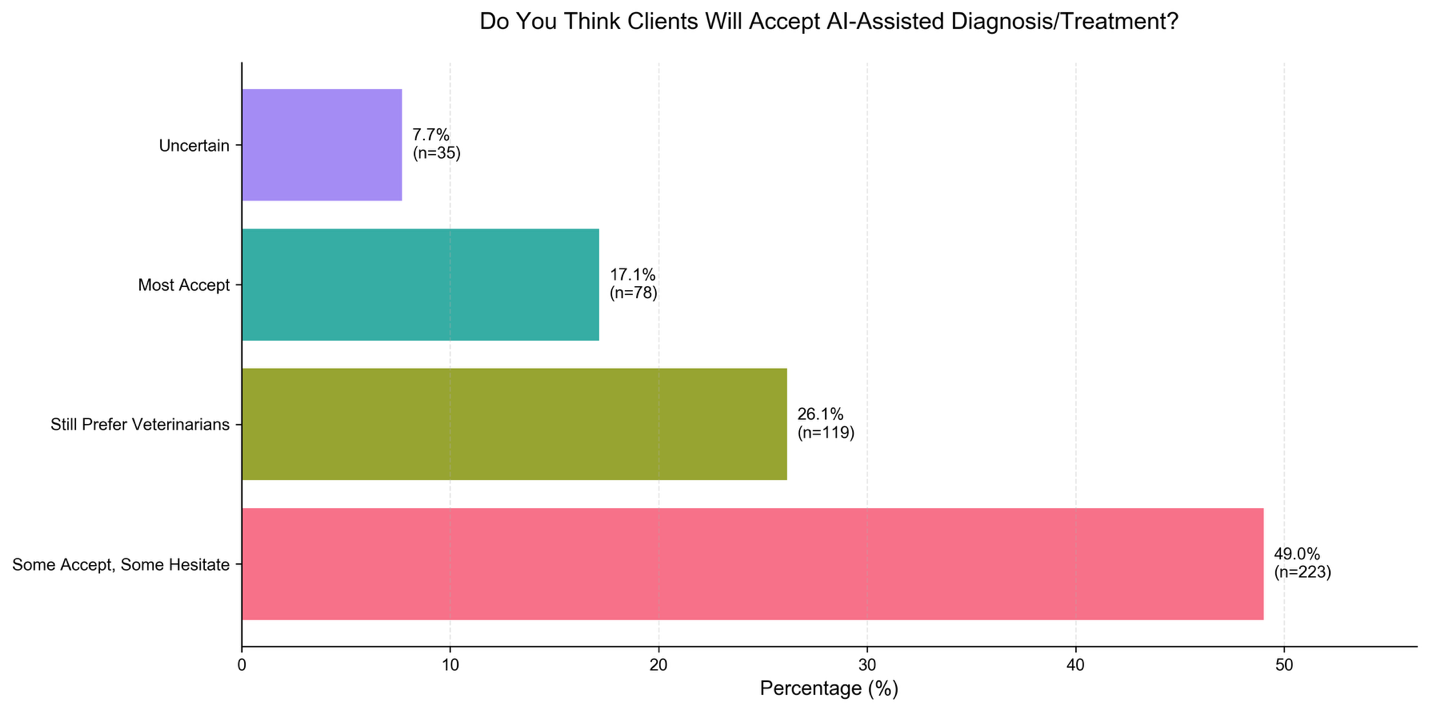

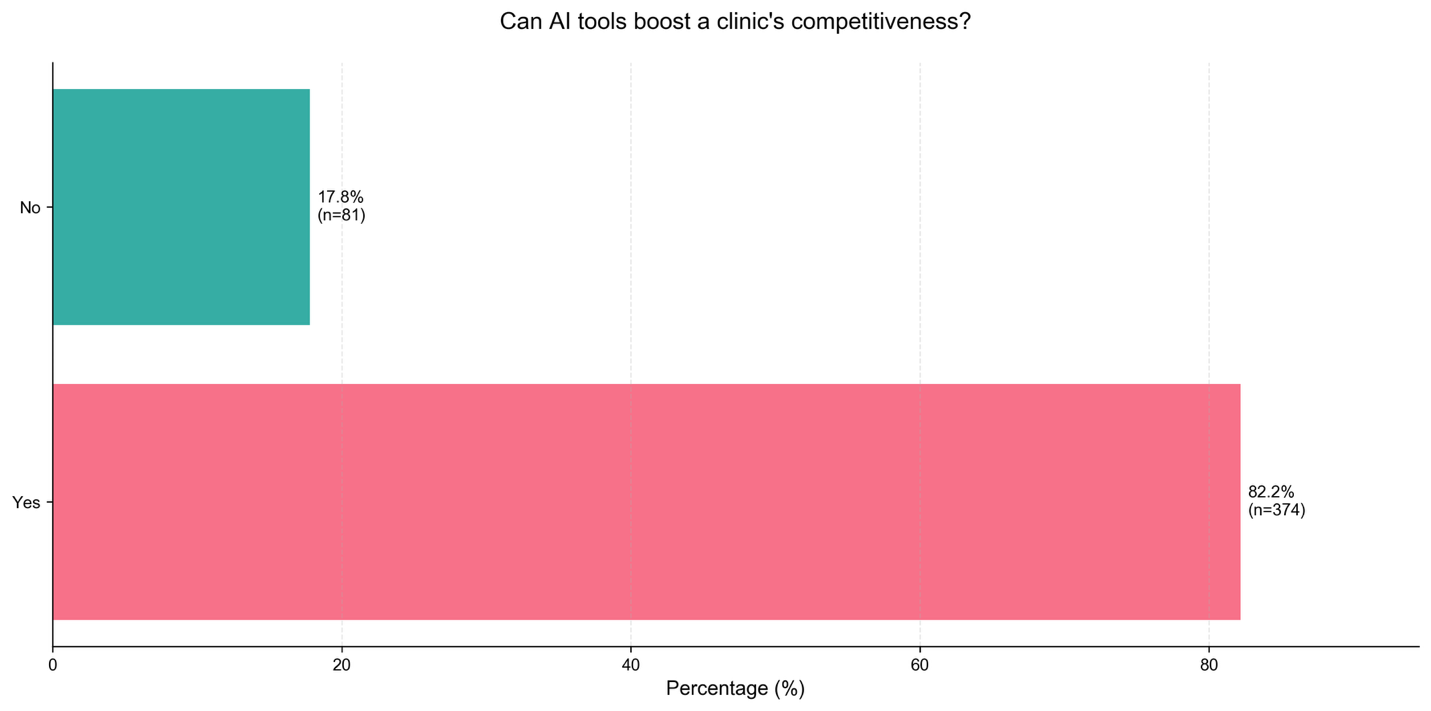

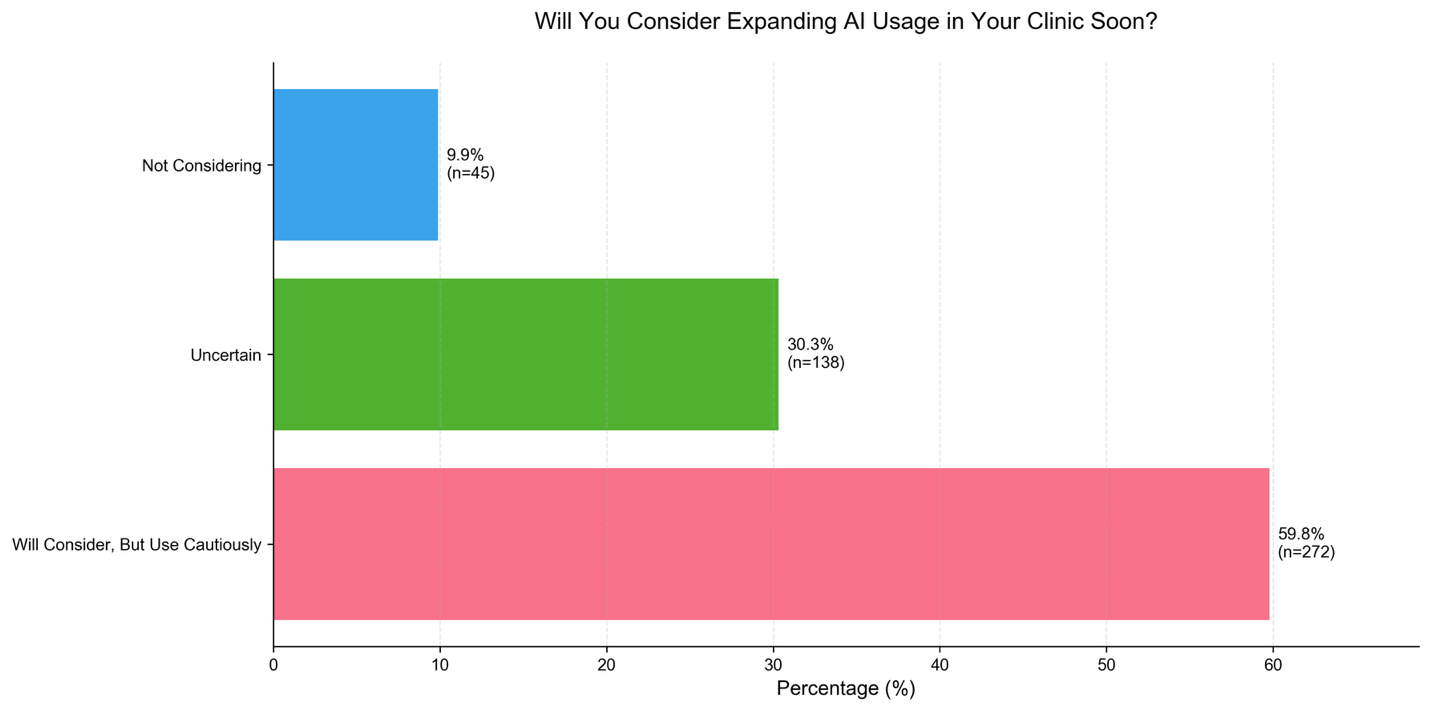

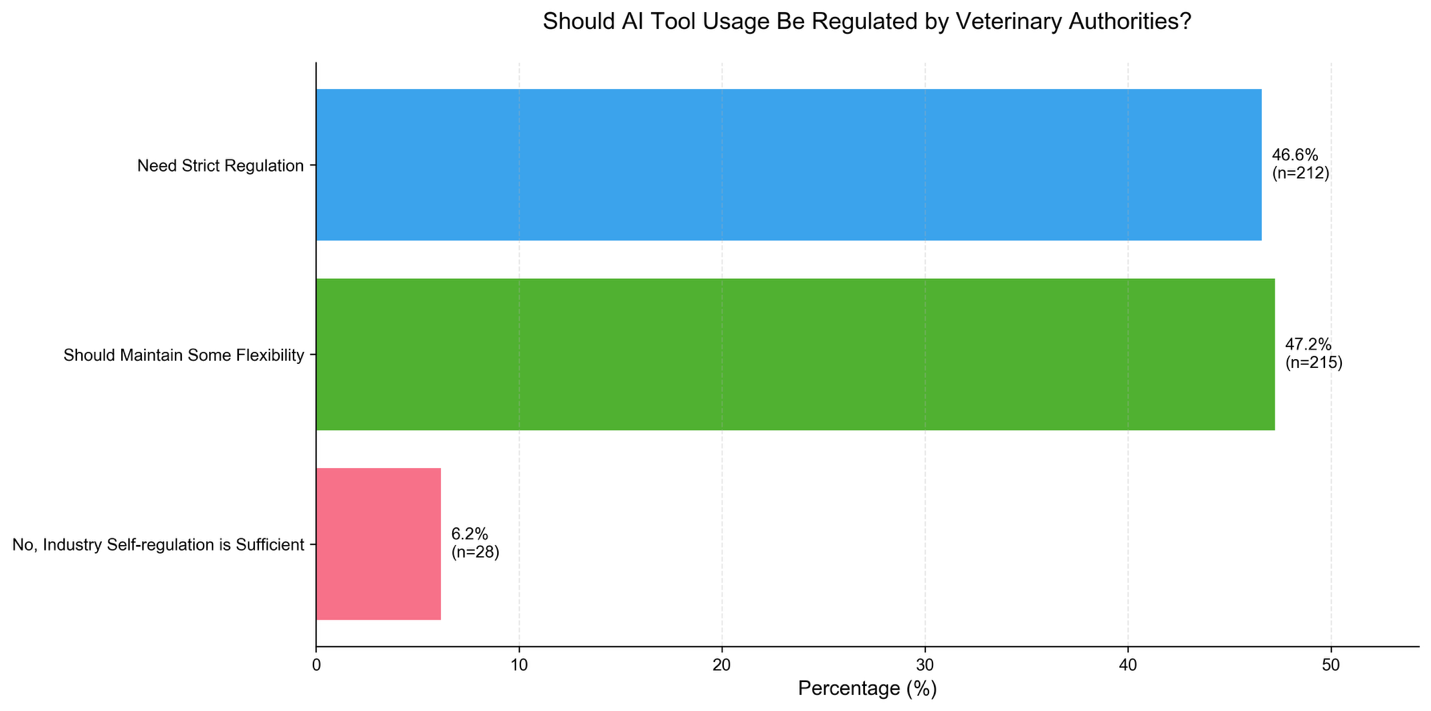

Supplement: Supplementary file 1 [file Table_1.DOCX]
